# Midwifery ethics in care, practice, and education: A scoping review

**DOI:** 10.18332/ejm/231691

**Published:** 2026-10-09

**Authors:** Kate Buchanan, Elizabeth Newnham, Michele Megregian, Maria Ekelin, Tahlia Johnson, Tita Stanek Zidaric, Ardelia Prastika, Sareh Dashti, Nakachew Amare, Stephanie Marriott

**Affiliations:** 1 School of Nursing and Midwifery, Edith Cowan University, Joondalup, Australia; 2 Flinders University, Adelaide, Australia; 3 School of Nursing, Oregon Health and Science University, Portland, United States; 4 Department of Health Sciences, Lund University, Lund, Sweden; 5 Faculty of Health Sciences, University of Ljubljana, Ljubljana, Slovenia; 6 Department of Midwifery, Faculty of Medicine, Universitas Airlangga, Surabaya, Indonesia; 7 Department of Nursing and Midwifery, Islamic Azad University, Mashhad, Iran; 8 School of Nursing and Midwifery, Asrat Woldeyes Health Science Campus, Debre Berhan University, Debre Berhan, Ethiopia; 9 International Confederation of Midwives, the Hague, the Netherlands

**Keywords:** ethics, relational ethics, midwifery ethics, scoping review, midwifery philosophye

## Abstract

**INTRODUCTION:**

Midwifery ethics is central to high quality, respectful and relational maternity care. To inform the update of the International Confederation of Midwives, International Code of Ethics for Midwives, this scoping review systematically maps how ethics in midwifery care, practice and education is characterized.

**METHODS:**

Joanna Briggs Institute (JBI) methods for a scoping review were used to guide the literature review process. The Population/Concept/Context (PCC) framework provided the boundaries for the review. Population: midwives, midwifery educators, midwifery researchers, midwifery students and women mainly receiving midwifery care. Concept: midwifery ethics. Content: midwifery care, practice and education. Searches of qualitative and quantitative literature, and discussion papers, spanning 2002–2025, were made using MEDLINE, CINAHL, PsycINFO, MIDIRS, Emcare, Google and citation chaining. Data were extracted using an iterative content analysis approach extracting key ethical characteristics. The protocol for this review was prospectively registered with the Open Science Framework.

**RESULTS:**

The search yielded 363 screened records, with 101 full texts assessed against inclusion criteria. Fifty articles were included in the review. Data was grouped to four interconnecting ethical concepts: Relational ethics emphasizes the importance of relationship, trust and emotional safety; the Practice of Ethics highlights care ethics attributes and accountability; the Ethical use of knowledge underscores evidenced based practice, reflexivity and sharing knowledge to equalize power; and Ethical leadership extends midwifery philosophical commitment to advocacy and reproductive justice.

**CONCLUSION:**

This review provides a contemporary map of midwifery ethics and foundational evidence to support the update to the global International Confederation of Midwives, International Code of Ethics for Midwives.

## INTRODUCTION

The International Confederation of Midwives (ICM) founded in 1922, is a global organization representing over 119 countries through member association^1^. Guided by the Professional Framework^2^ which centers on the Philosophy of Midwifery Care^3^, the ICM sets the global standards for the profession. Since at least 1993^4^, the ICM has had an International Code of Ethics for midwives^5^. The code has far-reaching influence on the practice, regulation and education of midwives^6^, therefore it is critical that the Code is grounded in evidence, demonstrating the principles that reflect midwifery philosophy, knowledge and practice; addresses documented ethical challenges; and aligns the code with best evidence. The authors of the review served as ICM co-convened experts in 2025–2026 to update the Code. To support the process of updating the Code, the group conducted a scoping review of the literature to inform the proposed updates.

Codes of ethics do not set out enforceable standards for professional behavior, as that is the role of the Code of Conduct^7^, but provides the moral and professional values on which the code of conduct is built; as codes of conduct are closely linked to law and legislation, these are not usually established internationally. A code of ethics, however, can provide guidance locally, nationally and internationally^5^. The aim of the ICM’s code of ethics for midwives is to provide such guidance for midwives and regulators globally^5^. While health professionals’ codes of ethics are grounded in the well-known bioethical principles of non-maleficence, beneficence, autonomy and justice, emerging scholarship positions care ethics as a central tenant to midwifery ethics as a more closely aligned framework for guiding the ethics of midwifery practice^8^. Increasing reports of dehumanizing and abusive care and birth trauma^9^ underscore the need for renewed ethical attention within the profession, including the ethical preparation of midwives during education^10-12^.

The working group sought to provide systematic examination of evidence that could inform the international Code of Ethics for midwives. The review therefore aims to map the scope, nature and use of ethical concepts within midwifery literature.

This scoping review examines how ethics is described, conceptualized and applied across midwifery care practice and education. Scoping reviews are a form of evidence syntheses that map the breadth of available evidence on a topic and can include primary research and non-empirical evidence such as discussion of theory^13,14^; important to this review, as there is much contemporary discussion of what should be included in midwifery ethics but less empirical evidence of midwifery ethics^6^. The scoping review was selected as it allows the team to map key ethical concepts rather than comparing and evaluating quality^14^. Scoping reviews can be used to clarify key concepts and identify characteristics or factors related to the concept. A preliminary search confirmed that relevant literature is available to meet the inclusion criteria. The existing literature related to midwifery ethics is conceptually dispersed, inconsistently described and spans theoretical descriptive and empirical scholarship. Therefore, the scoping review, conducted in accordance with the Joanna Briggs Institute methodology^13,14^, provides the most appropriate systematic approach for identifying the breadth of ethical concepts that shape how ethics is conceptualized in midwifery care practice and education. This comprehensive mapping is essential for informing the update of the International Code of Ethics for Midwives. The review question was: ‘How are midwifery ethics in midwives’ and student midwives’ care provision, practice, and education characterized and understood in the literature?’. The review objective was to systematically identify and characterize the key words, concepts, and ethical constructs within existing midwifery literature that may inform the development of a midwiferyspecific code of ethics.

## METHODS

### Ethical considerations

As this project was a literature review, this study did not involve direct engagement with human participants. The review was conducted in adherence with recognized principles of research with integrity, transparency and ethical scholarship.

A scoping review approach was chosen for this study as it aligned with the research objective to map the concept of midwifery ethics^14^. This scoping review was undertaken in accordance with the JBI methodology for scoping reviews and the Preferred Reporting Items for Scoping Reviews (Supplementary file PRISMA-ScR checklist^15^). Critical appraisal of included sources of evidence was not performed as the breadth of available evidence was the review focus^14^. The review team met regularly throughout the entire process, including data extraction, analysis, and presentation, to promote consistency and methodological rigor. Online meetings allowed the team to refine extraction decisions and maintain a shared understanding of the developing evidence. The protocol for this review was prospectively registered with the Open Science Framework on 18 September 2025, https://doi.org/10.17605/OSF.IO/752DH.

### Inclusion criteria

Articles published between 2002 and 2025 were included. Eligible sources comprised peer-reviewed primary research (all methodologies) and systematic reviews that provided a definition or description of ethics in midwifery care, practice, or education. While most grey literature was excluded, discussion papers that were peer reviewed were retained. Scoping reviews may include discussion papers when they contribute to the conceptual theoretical perspectives relevant to review questions^14^. Only English-language sources were eligible. Full-text items were included when they addressed midwifery ethics through a definition or description relevant to midwifery care and practice within the maternity context (antenatal, intrapartum, postnatal, abortion care, or sexual health), or when they described or defined midwifery ethics within an educational context (Table 1).

**Table 1:** Inclusion and exclusion criteria applied to study selection in the scoping review of midwifery ethics in midwives’ and student midwives’ care provision, practice, and education

*Inclusion criteria*	*Exclusion criteria*
Peer reviewed primary research (qualitative, quantitative, mixed methods) and systematic reviews that include definition or description of ethics in midwifery care, practice, and/or education Discussion papers, that were peer reviewed and develop conceptual and theoretical understandings English language available Full text available Includes definition or description of ethics in midwifery care and/or practice in the maternity context, including antenatal, intrapartum, postnatal, abortion care, and sexual health; or definition or description of midwifery ethics in an educational context Literature published between 2002 and 2025	Non-peer reviewed research Study protocols, commentaries, editorials, Languages other than English with no translation to English available Full text unavailable Ethics related to health professions and/or health professionals outside of midwifery Ethics described in educational settings outside of midwiferyspecific education provision Does not include detailed description or clear defining characteristics that contribute to understanding of midwifery ethics

### Exclusion criteria

Excluded sources included meta-analyses, study protocols, literature not in English without translation, and items for which full text was unavailable. Also excluded were materials relating to ethics in health professions outside midwifery or sources lacking sufficient detail or defining characteristics to support understanding of midwifery ethics (Table 1).

### Participants

Included participant groups were midwives, midwifery educators, midwifery researchers, midwifery students, and perinatal women receiving care from midwives. Participant groups from other health professions were excluded.

### Concept

The concept of interest was any definition or description of ethics in midwifery care and/or practice within the maternity context (including antenatal, intrapartum, postnatal, abortion care, and sexual health), or a definition or description of midwifery ethics within an educational context. Sources were excluded when they did not contain sufficiently detailed description or clearly defined characteristics contributing to an understanding of midwifery ethics.

### Context

Midwifery ethics were explored across hospital, homebirth, and midwifery group practice settings, as well as within educational environments within midwifery-specific programs.

### Types of sources

This scoping review considered for inclusion quantitative and descriptive observational study designs, including case series, individual case reports, and descriptive cross-sectional studies. Studies that focused on qualitative data were considered, including (but not limited to) phenomenology, grounded theory, ethnography, qualitative description, action research, and feminist research. Systematic reviews that met the inclusion criteria were also considered for inclusion, depending on the research question. Discussion papers were included, if they developed conceptual or theoretical understanding of ethics and midwifery related to the review question.

### Search strategy

The Population/Concept/Context (PCC) was used to develop the review search terms, as recommended by Peters et al.^14^. A three-step approach was adopted for the review. Preliminary searches in MEDLINE were first conducted to identify articles related to midwifery ethics; search strings were subsequently tested and refined as needed. The second step involved using appropriate search strings to search the databases MEDLINE, CINAHL, PsycINFO, MIDIRS, and Emcare. Then, both ascendancy and descendancy citation searching was carried out to identify additional potential evidence sources.

The search strategy was refined through iterative preliminary database searches. A broad initial search (e.g. title and abstract search: ethic*; MeSH term: midwifery) was gradually refined in subsequent searches, ensuring that the population, concept, and context of the review were focused and key publications remained in the search results. The search strategy was reviewed and discussed by the research team.

### Other search strategies

Citation searching (ascendancy approach) of sourced literature for the review was employed. Text and opinion papers were considered for inclusion in this scoping review. The information sources were the following databases: CINAHL (EBSCO), MEDLINE (EBSCO), PsycINFO (EBSCO), Emcare (Ovid), MIDIRS (Ovid), and the search engine was Google. The MIDIRS database was used to source reports and/or policy documents (in addition to peer reviewed research). Google search was used to source codes of ethics from relevant professional bodies; the first 100 hits from Google were independently screened by two reviewers.

### Query strings

The query strings used for each database were as follows:

CINAHL: MM “Midwifery+” AND XB (midwi* AND ethic* AND (car* OR practic* OR educat*))

MEDLINE: MM “Midwifery” AND XB (midwi* AND ethic* AND (car* OR practic* OR educat*))

PsycINFO: DE “Midwifery” AND XB (midwi* AND ethic* AND (car* OR practic* OR educat*))

MIDIRS: (midwi* AND ethic* AND (car* OR practic* OR educat*)).ab.

Emcare: (midwi* AND ethic* AND (car* OR practic* OR educat*)).ab.

Google: midwifery AND ethics AND codes

### Study/source of evidence selection

References from database searches (n=405) (MEDLINE 96; MIDIRS 91; CINAHL 76; PsycINFO74; Emcare 68) were imported to Covidence for screening and automatically deduplicated (118 removed), after which 287 articles remained. After a pilot test, these titles and abstracts were screened following independent blinded double screening procedure. The two screeners were guided by the research objective, review question, and inclusion criteria. Following title and abstract screening, citation searching was conducted using both descendancy and ascendancy approaches. A total of 852 citations were screened in Google Scholar through descendancy searching, yielding 52 references for import into Covidence, while 1118 reference list entries were screened through ascendancy searching, yielding 24 references. Overall, 76 references were imported to Covidence from citation searching. The combined figures of 287 and 76 resulted in a total number of 363 references screened. A total of 101 publications progressed to full text review after 262 were excluded at the title and abstract stage. Full texts were then assessed in detail against the inclusion criteria by two independent reviewers and reasons for exclusion were recorded. Any disagreements that arose between the reviewers were resolved through discussion and in consultation with a third reviewer. The results of the search and the study inclusion process are presented in a PRISMA-ScR flow diagram (Figure 1).

**Figure 1:**
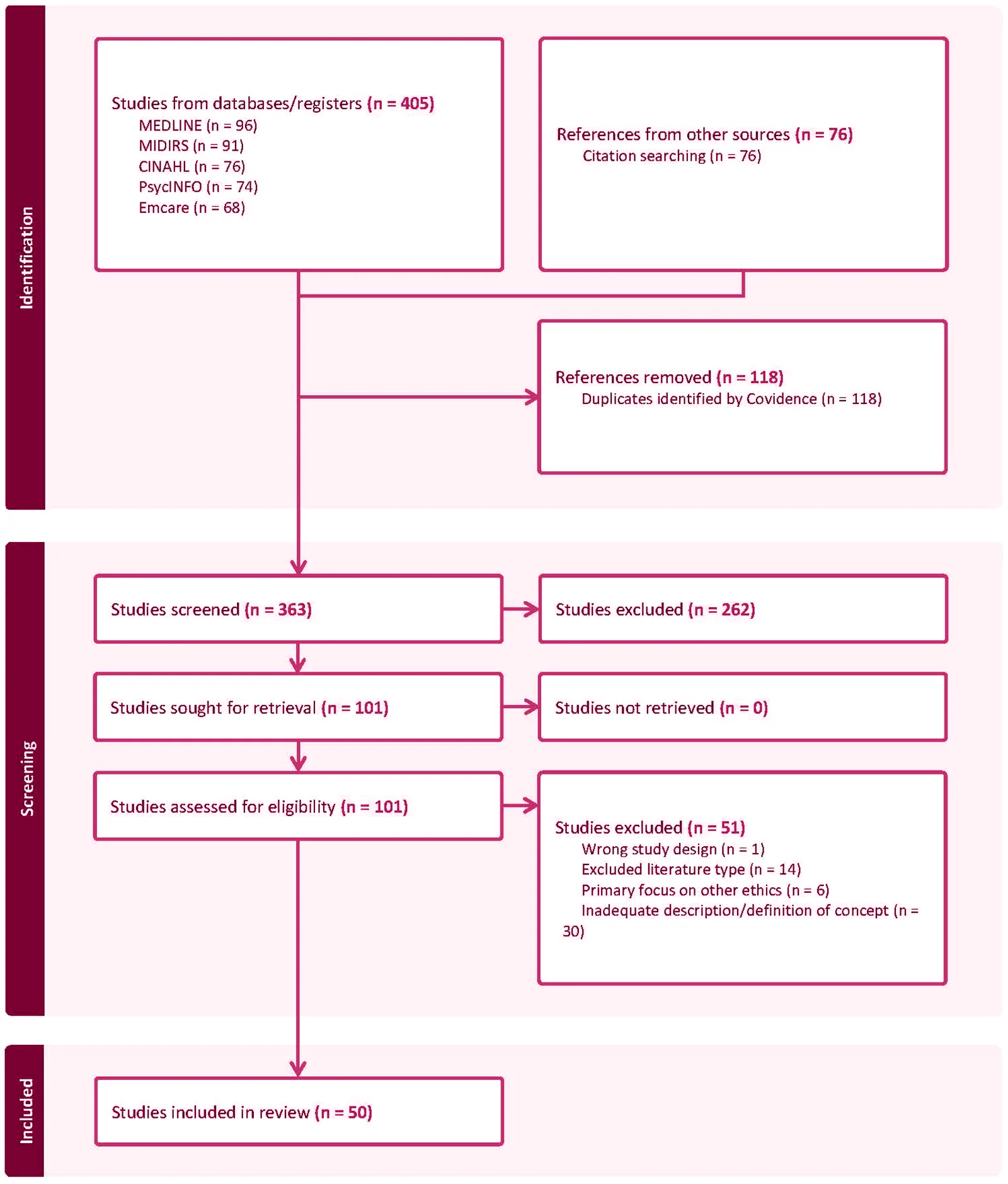
PRISMA-ScR flow diagram of study selection for the scoping review of midwifery ethics in midwives’ and student midwives’ care provision, practice, and education, 2002–2025

### Data extraction

Data were extracted from articles included in the scoping review by all authors using a data extraction tool developed by the reviewers, based on the JBI data extraction recommendations^13^. Two data extraction tables were developed. Table 2 included standardized JBI data extraction: Author, year, country, research aim, design, participant characteristics and main findings related to midwifery ethics relevant to the review question. Supplementary file Table 1 included key ethical concepts addressed in each article, coding these concepts into keywords, and indicating how each keyword or concept aligned with, or related to, specific statements within the Code of Ethics. An *a priori* protocol and draft extraction form was developed and refined after the first round of coding (pilot tested) and review with all authors, which helped identify additional information requirements and clarified issues.

**Table 2:** Characteristics of selected articles, focusing on research aim and design, participants, context, and main findings related to midwifery ethics (N=50)

*Author Year Country*	*Research aim and design*	*Participants, characteristics, total number and context*	*Main findings related to midwifery ethics*
Berkiten-Ergin et al. 2013 Turkey^17^	**Aim:** To identify the professional values of midwifery in Turkey, particularly those shaped by local cultural influences, in order to address the lack of defined independent roles, ethical values, and moral codes specific to Turkish midwifery. **Design:** Qualitative focus group study using in-depth interviews across three meetings, with thematic analysis of transcribed recordings and notes.	**Participants:** Nine midwives with a mean age of 28 years and mean professional experience of 7 years; five were high school graduates, and four had university-level training. **Context:** Participants were selected from three hospitals (two state hospitals and one university hospital) in Kocaeli, an industrial city in Turkey with a diverse population representing the country’s social structure.	**Findings:** Turkish midwives identified key ethical values in midwifery that aligned closely with international standards but showed some cultural nuances. Professional competence emerged as the top priority, emphasizing the need for midwives to possess sufficient knowledge and skills to ensure safe care, followed by trustworthiness, responsibility, maximum benefit (and minimum harm), respect for privacy, equality, and empathy. Participants classified characteristics of a good midwife into professional (competence, informing parties properly, respecting human dignity), personal (trustworthiness, patience, altruism), and interpersonal (empathy, effective communication), underscoring that these traits support ethical practice.
Buchanan et al. 2025 Australia^6^	**Aim:** To describe midwives’ perceptions and experiences of ethics in midwifery practice, and to determine the crucial features of midwifery ethics. **Design:** A two-round Delphi approach was employed, conducted through a feminist standpoint theoretical lens. Round One involved qualitative data collection via open-ended survey questions, analyzed using descriptive thematic analysis. Round Two involved ranking and commenting on statements derived from Round One data to reach consensus (80% agreement threshold).	**Total number:** 50 midwives from 21 countries. **Context:** Employment context almost 60% in public hospitals, 6% in private hospitals, 34% in midwifery models of care, and midwifery practice role.	Midwifery ethics is based in Midwifery Philosophy (Domain 1), which prioritizes woman-centered care, reproductive justice, and solidarity with women’s autonomy. underpinned by a discrete knowledge base supporting physiological processes, full scope of practice with accountability, and integration of evidence with women’s contextual knowledge. Midwives foster trust-based partnerships through continuity and respect. (Domain 2) which means building trust-based partnerships through continuity, understanding individual contexts non-judgmentally, and fostering self-determination via unbiased information. (Domain 3), commitment to human rights and reproductive justice, holistic care respecting diversity for equity and safety, centering on birthing people’s values, and self-awareness of personal values to maintain impartiality. Having integrity to stand up and challenge those with stronger or different opinions, give a voice to women. (Domain 4). Which means advocating for women’s rights against systemic barriers, including navigating power imbalances and supporting informed choices even when challenging.
Buchanan 2025 Australia^10^	**Aim:** To understand students’ perception of ethical issues and to use these findings as foundational information with which to create an ethical deliberation tool for practice. **Design:** Qualitative Content Analysis, guided by a critical realist feminist theory. The assignments were based on personal experiences of ethical situations during clinical placements in midwifery programs.	**Participants:** 21 (data from twenty-one ethics-focused reflective assignments). **Context:** Participants included twelve postgraduate midwifery students and nine undergraduate midwifery students They were in their final semester in 2023 or had graduated the semester before in 2022, from Edith Cowan University in Western Australia, Australia.	Three themes were created which captured midwifery students experience of ethics on clinical placement: ‘Bearing witness and feeling moral distress’; ‘Relationship with the woman fostered ethical awareness’ and ‘Learning to advocate’. Midwifery students identified ethical issues during practical placement related to consent, interventions, insensitive practice, and overuse of technology, but felt powerless to act, which caused subsequent moral distress.
Buchanan et al. 2023 Australia^18^	**Aim:** To explore women’s experiences of maternity care from an ethical perspective. **Design:** Feminist Participatory Action Research (FPAR).	**Participants:** 19, all of whom were cis-gender women who had experienced a midwifery model of care in Western Australia. They were educated, partnered, employed, and predominantly Caucasian (born in Australia or of European descent). All had prior experiences with medical models or interactions with the medical system during pregnancy or birth.	A unique ethical perspective was described by the participants. The central theme: ‘Radical desires: Individuals values and context’ placed the woman at the center of the care, in determining what is ethical. The findings emphasize that midwifery ethics should prioritize relational, individualized care to counter medicalization and disrespect, integrating feminist care ethics theory to enhance everyday practice and reduce harm in maternity systems. A conceptual model Woman-centered ethics is offered to enhance everyday ethical midwifery care. Authoritarian ethics involved uneasy alliances (poor relationships), opaque information (biased/withheld knowledge), and ‘saving women from themselves’ (control removing agency), leading to surrendered power (disembodiment, trauma). Woman-centered ethics involved midwifery solidarity (strong relationships), transparent knowledge (unbiased information), and physiology supported (agency/choice), leading to claiming power (embodiment, empowerment).
Buchanan et al. 2022 Australia^19^	**Aim:** Determine how care ethics is used amongst health professions and to collate the information in data charts to create a care ethics framework and definition for midwifery practice. **Design:** Scoping review conducted according to the Preferred Reporting Items for Scoping Reviews (PRISMA-ScR) and Joanna Briggs Institute (JBI) methodology, involving database searches (CINAHL, MEDLINE, PsycInfo, PubMed) in September 2019 and July 2021, limited to qualitative studies in English from 2010 to 2021.	As a scoping review, analyzed 12 included qualitative studies involving health professionals such as nurses, doctors, physiotherapists, and social workers. **Context:** Focused on health care practice settings, including elderly residential care, community home support, acute care, palliative care, and decision-making processes in various health professions.	Care ethics was referenced in health care practice in four broad categories relationship, caring practices, context and attention to power. Care ethics use by health professionals enhances ethical sensitivity. A working definition of care ethics for midwifery practice was proposed: Care ethics recognizes that care is a universal human experience, founded on relationship based on presence, trust, and respect, forged on knowing the person at the center of care. The practice of care is holistic, attentive, responsive, responsible, and competent, considering the richness and complexity of the individual’s socioemotional context while equally valuing other ways of knowing. It acknowledges the asymmetry of caring relationships and requires attention to power imbalances, with only the person being cared for determining what constitutes ethical care. This definition positions care ethics as a supplementary paradigm to bioethics, potentially reducing mistreatment in maternity care through reflective, relational practices.
Buchanan et al. 2022 Australia^8^	**Aim:** To determine whether midwifery models of care demonstrated care ethics. **Design:** Template analysis.	**Participants:** 9 childbearing women who had had midwifery continuity of care. **Context:** Homebirth or family birth center models of care.	Continuity of care, particularly with same caregiver, essential to ethical care whereby individualized care relationships are essential for women to actualize autonomy and choice, levels power and that the socio-cultural contexts of birth are significant. Ethical midwifery focuses on promoting physiologic birth and trust in the inherent processes of labor/birth same philosophy of birth, respected her agency and would aim to strengthen her capabilities the re-cognition of birth as a rite of passage into well motherhood. Ethical midwifery care, power differentials are flattened through emphasis on transparency in information exchange and respect for decisions made. Ethical care was demonstrated when they women had the power to make decisions and to exercise agency Care ethics may be an alternative ethical framework to the principles model for perinatal and midwifery care.
Carvajal et al. 2024 Chile^20^	**Aim:** To elucidate how Chilean midwives have (re)defined their professional identity to include abortion care after the enactment of the abortion law. Constructivist grounded theory Online interviews. Constant comparison methods for codes. **Design:** Constructivist Grounded theory methodology.	**Participants:** 15 midwives 9 provided abortion care 13 women 5 atheists 2 conscientious objectors 1 identified as an ‘ethnic group’ 4-42 years’ work experience Median age 33 years **Context:** Examination of midwifery values/beliefs after abortion was decriminalized (in 3 circumstances) in 2017.	Findings highlight that the inclusion of induced abortion care in Chile has created tension in two aspects of midwives’ identity. While Chilean midwives see themselves as “women-centered” professionals, they also acknowledge their roles towards “protecting life””. Furthermore, abortion puts into tension the woman-centered ethos of midwives and their relationship with the fetus. There is a strong sense among interviewees that midwifery has been historically about working with and protecting life. When midwifery is perceived as naturally more inclined towards life, witnessing death becomes particularly challenging, irrespective of midwives’ moral position regarding abortion and beyond abortion care: This variability in midwives’ response to the abortion law seems to relate to how strongly they adhere to being ‘woman-centered’ professionals and their moral positioning regarding abortion.
Ergin et al. 2013 Turkey^21^	**Aim:** To help professionalize midwifery and to determine the national ethical rules and codes of midwifery. **Design:** In-depth interviews with 12 participants to develop questionnaire. Descriptive values to analyze demographics and each questionnaire value 2011.	**Participants:** Interview: 12 participants (9 midwives, 1 supervisor, 2 specialist academicians, 1 reporter) Questionnaire: N=1067 **Context:** Midwives working in delivery rooms and obstetric clinics in hospitals in 12 regions.	The five professional values that the participants ranked as most important were, in order, (a) caring for mother-child health, (b) being responsible, (c) having professional competence, (d) having respect for privacy and (e) being reliable. The values that the participants ranked as least important included, in order, sympathy, goodwill, altruism, inner peace, humility and sincerity. The midwifery ethical codes that the participants ranked as most important were, in order, avoiding conflicts of interest, protecting privacy, avoiding deception, reporting faulty practices, seeing the mother and newborn as separate beings and preventing harm. The suggested additions made by the participating midwives to the ethical code list included (a) being open to professional developments and following them, (b) giving priority to professional ethics and protecting them, (c) being disciplined in professional practice, (d) being aware of her powers and limitations, (e) valuing patients and letting them know that they are valued and (f) being able to warn her colleagues if something goes wrong.
Firoozehchian et al. 2022 Iran^22^	**Aim:** Clarifying clinical competence, design a prerequisite for designing a psychometric and valid tool to evaluate the clinical competence of Iranian undergraduate midwifery students. **Design:** Qualitative and conventional content analysis of interviews, developing codes and themes.	**Participants:** N=24 7 undergraduate students 9 faculty members 7 working midwives 1 obstetrician **Context:** Midwifery and nursing schools and hospitals and health centers affiliated to Tehran and Guilan universities of medical sciences in Iran.	**Category 1:** Ethical and professional function in midwifery. 1-1 Integration of science and practice in midwifery; 1-2 compliance with ethics and regulations in providing midwifery care; 1-3 Improving the independent function of midwifery; 1-4 evidence-based practice. **Category 2:** Holistic Midwifery Care. 2-1 involving women and their families in providing midwifery and reproductive and sexual health services; 2-2 providing education and support to women and their families for promoting reproductive and sexual health. **Category 3:** Effective interaction. 3-1 using communication skills to provide midwifery and reproductive and sexual health services; 3-2 effective and coordinated communication with other people providing midwifery and reproductive and sexual health. **Category 4:** Personal and professional development. 4-1 Feeling responsible and committed to learning for yourself and your peers. 4-2 accountability in providing midwifery and reproductive and sexual health services.
Fontein-Kuipers et al. 2018 the Netherlands^23^	**Aim:** Explore dilemmas, conflicting values, and underlying factors that Dutch midwives experience when they find it difficult to confirm to, or to accommodate women’s care needs. **Design:** Narrative inquiry - interviews. Identification of themes.	**Participants:** Midwives providing midwifery led care N=11. **Context:** Community based midwifery proving midwifery led care.	The first theme describes the midwife’s experienced dilemma from simultaneously wanting to be loyal to the woman’s needs, consisting of her wishes and expectations to achieve or accommodate optimal experiences and to be loyal to guidelines, evidence, and collaborative relationships with other professionals. This theme [responsibility] describes the dilemma that the midwife experiences when it needs to be determined who is responsible and accountable for choices, decisions, and actions during the care process. The dilemma originates from simultaneously feeling responsible for doing the woman justice as an individual, being honest, and “being good” to her and to be regarded as a responsible and trustworthy professional by colleagues. The third theme [selfhood] describes the midwife’s dilemma caused by a conflict between the woman’s and the midwife’s levels of assertiveness or compliance. biomedical model which is reliant on evidence and knowledge, and a humanistic and holistic model which values physical and psychosocial well-being of women.
Foster et al. 2022 Australia^24^	**Aim:** To explore Australian midwives experience and consequences of moral distress. **Design:** Qualitative descriptive study using semi-structured interviews (in depth interviews), with thematic analysis of verbatim transcribed audio recordings conducted using NVivo 12.	**Participants:** N=14 registered midwives. **Context:** Participants (all female) represented rural, tertiary, and private practice settings across five Australian states and territories.	The study identified moral distress as an ethical issue in Australian midwifery practice, occurring when midwives faced situations that conflicted with their professional and personal values. Three main themes emerged: moral compromise, where midwives felt compelled to act against what they believed was right; moral constraints and ethical uncertainty, often linked to hierarchical and restrictive workplace cultures; and professional and personal consequences, including ongoing emotional distress and ethical strain.
Foster et al. 2022 Australia^25^	**Aim:** To understand the concept of moral distress in the context of midwifery practice, describing the attributes, antecedents and consequences. **Design:** Concept analysis using Rodgers’ evolutionary framework, forming the first stage of a sequential mixed-methods study. A literature review of eight articles was conducted, with data analysed using NVivo 12.	Three databases were searched: PsycINFO, OVID Medline, and OVID Emcare. Of the eight articles included in this concept analysis, five were qualitative studies, two were quantitative studies, and one used a mixed-methods design.	Three core attributes were identified: moral actions and inactions, conflicting needs and negative feelings/emotions. The antecedents of clinical situations, moral awareness, uncertainty and constraint were identified. Consequences of moral distress include adverse personal professional and organizational outcomes. A model case depicting these aspects is presented. A midwifery focused definition of moral distress is offered as ‘a psychological suffering following clinical situations of moral uncertainty and/or constraint, which result in an experience of personal powerlessness where the midwife perceives an inability to preserve all competing moral commitments.
Getu et al. 2025 Ethiopia^26^	**Aim:** To explore the ethical dilemmas experienced by midwives in their professional practice. **Design:** Qualitative phenomenological study using interpretative phenomenological analysis with in-depth, face-to-face semi-structured interviews. Data were analyzed using thematic analysis supported by ATLAS.	**Participants:** N=14 midwives. Participants had a minimum of two years of clinical experience and were practicing in northwestern Ethiopia. **Context:** Referral hospitals within the Amhara regional state. Eligibility criteria included direct experience with ethical dilemmas in clinical practice, availability during the study period, and provision of informed consent.	The findings showed that midwives frequently faced ethical dilemmas in their daily professional practice. These dilemmas reflected complex interactions between personal beliefs, professional ethic, institutional structures, and respect for patient rights. Analysis of the data identified five main themes of ethical dilemmas, supported by ten sub-themes and illustrated through participants’ narratives. The identified themes included cultural and religious views, confidentiality and communication, autonomy versus beneficence, unsafe work environments, and professional role conflict.
Hallgren et al. 2005 Sweden^27^	**Aim:** To describe how prospective mothers, fathers, and midwives relate to one another during childbirth, and to reflect on these interactions in relation to parents’ previously reported experiences and Løgstrup’s theory of relational ethics. **Design:** Qualitative content analysis based on observations, interviews, and video-recorded interactions.	**Participants:** N=4 couples and 9 midwives. **Context:** The study was conducted in antenatal clinics and on a delivery ward in a midsize public hospital in Central Sweden.	The findings highlighted both ethical and unethical ways in which midwives related to women and their partners during childbirth. Results of the analysis showed that ethical practice was reflected in relational behaviors characterized by presence, responsiveness, and respect, while unethical practices involved distance, lack of attentiveness, or failure to acknowledge parents’ vulnerability. These findings were interpreted in light of Løgstrup’s relational ethics, emphasizing the ethical responsibility inherent in midwives’ relationships with women and their partners during childbirth.
Haseli et al. 2024 Iran^28^	**Aim:** To explore the experiences of midwifery students regarding threats to women’s dignity during childbirth. **Design:** Qualitative study with an exploratory design, based on in-depth interviews and content analysis using MAXQDA software.	N=32 midwifery students. **Context:** The study was conducted at the Faculty of Nursing and Midwifery, Kermanshah University of Medical Sciences, Iran. Participants were midwifery students from a range of academic semesters who were in at least the fifth semester of a bachelor’s degree program or higher and had clinical experience in labor and delivery.	The findings revealed significant ethical concerns related to threats to maternal dignity during labor and birth, as perceived by midwifery students. Four main themes, encompassing sixteen sub-themes, were identified: professional incompetence, abuse of power imbalance, an exclusive focus on physical health with neglect of mental well-being, and structural issues within the system These findings highlight ethical challenges that undermine respectful, dignity-centered midwifery care and indicated that inadequate midwifery professional competence represents a major ethical concern, as limited awareness of professional ethics and reliance on outdated care practices contribute to non-evidence-based interventions, the normalization of disrespect, and the justification of violence during childbirth as routine care.
Hazal and Çalım 2022 Turkey^29^	**Aim:** To describe the ethical dilemma experiences of midwives working in delivery rooms in Turkey. **Design:** Qualitative phenomenological. Semi-structured, in-depth Audiovisual recorded interviews.	N=13 midwives, all with 2+ years of experience.	Differences in ethical approach (28) – mainly experience made for easier decision making & difficult decisions were upsetting – with feelings of helplessness. Four themes were identified: differences of approach to the birth process (105) – mainly unnecessary medicalization hospital management (86) – mainly hierarchical (doctor or management) pressures, unable to preserve dignity and lack of resources. Communication (21) – mainly conflict with colleagues. Ethical dilemmas faced by these midwives while working in delivery rooms were mainly caused by hierarchy - Limitations by medics to the scope of practice of a midwives seen as a threat to midwifery ethics.
Honkavuo 2022 Finland^30^	**Aim:** Research Question 1: What are the midwifery students’ experiences on support for ethical competence during midwifery education in clinical midwifery care practice? Research Question 2: How have the ethically challenging situations been carried out during clinical midwifery care practice? **Design:** Qualitative, explorative and descriptive design with inductive nature.	**Participants:** N=9 midwifery students. All female. **Context:** Students from three educational institutions at university level that educate midwifery students.	Midwifery students’ formation towards ethical competence is connected to a caring culture that permeates ethos, caring support and understanding from the educational institution and healthcare organizations, where the students meet the challenging world of clinical midwifery care. Main theme is a human and caring factor in the midwifery students’ process on ethical competence. Four subthemes: supporting through trust and responsibility – teachers, supervisors, peers. supporting through dignity and respect – of diversity in women/gender-diverse people supporting through truthfulness and justice – of the taught theoretical content supporting through dialogue and reflection – discussions in classroom, practice & with peers.
Khajehpour et al. 2024 Iran^31^	**Aim:** To aimed to assess the characteristics of hospital ethical climate in delivery ward of educational hospitals in southeast Iran. **Design:** Data collected using a demographic survey + the Hospital Ethical Climate Survey (HECS).	**Participants:** N=240 – midwives, midwifery students, midwife faculty. **Context:** Self-reporting questionnaire consisting of five subscales including relationship with peers, patients, managers, hospital, and physicians.	Olson’s Hospital Ethical Climate Survey, completed using the self-report method. The mean ethical climate in the midwifery group (3.82 ±0.63 out of 5) was higher than in the instructors’ and students’ groups. The lowest mean score obtained from the ethical climate questionnaire of participants was associated with the inability to use their experiences in the delivery ward. The lowest mean of ethical climate from the midwives’ point of view is the Physicians’ dimension and the patient’s dimension from the instructors’ point of view. The highest mean score belonged to the ethical climate of the supervisors. According to the results of the present study, it is suggested to implement protective laws to support the higher independence of midwives to improve the ethical climates by using their experiences in the delivery department.
Khatoon et al. 2025 Iran^32^	**Aim:** To examine the effect of the Dilemma Method of Moral Case Deliberation* on the knowledge and practice of midwives in the field of respectful maternity care. **Design:** Cohort Intervention (dilemma method of MCD) was conducted in groups of four or five midwives, with 3 sessions over a two-week period.	**Participants:** N=46 midwives. **Context:** 2 hospitals, 60km apart – similar size & demographics. One hospital control group (23), the other hospital intervention group (21) All participants undertook a pre- and post-intervention Midwives’ Knowledge and Practice Scale on Respectful Maternity Care (MKP-RMC).	The intervention group showed a significantly greater increase in knowledge scores from pre-test to post-test compared to the control group (p<0.001). The intervention group had a significantly higher mean change score in practice self-assessment and practice peer evaluation, from pre-test to post-test, than in the control group (p<0.001). Also mention in discussion about impact of power/ hierarchy/ medics on ethics. ‘A difference of opinion between a physician and a midwife can create a dilemma. The power hierarchy within medical teams can create challenges for midwives when resolving conflicts with doctors who hold higher positions. This can lead to stress and inappropriate interactions with patients.
Kiani et al. 2025 Iran^33^	**Aim:** This study aims to design and evaluate the psychometric properties of a tool to measure Adherence to the Code of Ethics in Midwifery (ACEM). **Design:** The construct validity was assessed using Exploratory Factor Analysis (EFA) on 200 samples through a cross-sectional study.	**Participants:** 200 midwives **Context:** Midwives working in various departments (public hospitals, private hospitals, health centers, university faculty members, university staff, research centers, headquarters, and private offices) across four regions of Iran: North, South, East, and West.	ACEM-42 is a valid and reliable tool to assess adherence to codes of ethics in midwifery practice. The tool included these topics: Professional commitment – competent, awareness of ethical responsibility, respect dignity, Service – Evidenced based practice, adheres to law, safety, responds to crisis, Women-centered care – privacy, confidentiality, empathy, respect, provide information, quality care, woman’s wishes, time, non-discrimination. Colleagues – cooperation and respect, reports non -compliance, Self – training self-monitoring /advancing the profession.
MacLellan 2014 United Kingdom^34^	**Aim:** Of this study was to examine the ethical foundations of midwifery practice using the feminist Ethics of Care framework and explore how institutional structures influence childbirth experiences and maternity care. **Design:** Comprehensive literature review.	**Participant:** Women receiving childbirth care and midwives working in hospital and educational settings, emphasizing women’s care experiences and midwives’ ethical challenges. **Context:** The review examined midwifery practice, maternity care institutions, and feminist ethics, focusing on ethical dilemmas, woman-centered care, and institutional influences on childbirth experiences.	Women during childbirth value relational aspects of care. The Ethic of Care approach equalize the relationship between midwife and woman, enabling relationship building and helping midwives fulfill their professional responsibilities. Ethic of Care as a feminist ethical framework that centers relationships, and mutual respect. - Relational balance between institutional demand and women’s needs. - Midwifery identity and practice being compromised without institutional acknowledgment of a distinct ethical approach.
Marsh et al. 2020 United Kingdom^35^	**Aim:** Midwives’ experiences of moral distress when providing care to women whose babies were removed at birth and gives valuable insight into an issue nurses and midwives encounter in their profession. **Design:** The study used a qualitative narrative inquiry design incorporating photo-elicitation techniques. Data were collected through face-to-face interviews with mothers and focus group discussions with midwives.	**Participants:** Mothers whose babies were removed at birth and midwives involved in providing their care. Twelve participants took part in the study, including four mothers and eight midwives. **Study context:** Maternity care, specifically exploring moral distress experienced by midwives when caring for women whose babies were removed at birth within contemporary clinical practice.	The study particularly focused on midwives’ emotional and professional experiences of moral distress. Midwives who care for women whose babies are removed at birth report it as one of the most distressing areas of contemporary clinical practice. Furthermore, they report feelings of guilt, helplessness and betrayal of the midwife–mother relationship. Many of the midwives in this study state that these experiences stay with them for a long time, far more than more joyful aspects of their role. Moral distress as a central ethical concept experienced when midwives’ professional and emotional values conflict with institutional or legal requirements.
Martín-Badia et al. 2021 Spain^36^	**Aim:** To explore midwives’ experiences in order to describe the ethical perspectives of obstetric violence and to examine how malpractice and violence in obstetric care affect key American and European bioethical principles. **Design:** The study used a qualitative phenomenological design.	**Participants:** A total of 24 midwives participated in the study. The participants were midwives who shared their experiences and perspectives regarding obstetric violence and ethical issues in maternity care. **Context:** Obstetric and maternity care.	Obstetric violence infringes on the main bioethical principles (non-maleficence, beneficence, autonomy, justice, vulnerability, dignity, and integrity). Beyond whether it is called violence or not, what matters from an ethical perspective is that, as long as women have such negative experiences during pregnancy and childbirth, obstetric care needs better humanizing. Humanization of care: Ethical midwifery practice requires recognizing women as biopsychosocial beings and promoting respectful, relationship-centered care.
Meddings and Haith-Cooper 2008 United Kingdom^37^	**Aim:** To critically examine the application of Gillon’s four-principles model of bioethics in midwifery practice, particularly in the context of caring for women from different ethnic, cultural, and linguistic backgrounds. **Design:** Theoretical and case-based ethical analysis.	**Participants:** Specific to Pakistani Muslim origin in the UK A case is presented. **Context:** Language and cultural barriers, that affect midwives providing ethically appropriate care to women of Pakistani Muslim origin.	Difficulties applying western bioethical principles - Autonomy, Beneficence and Non-maleficence, and Justice in cultural care Midwives may have difficulty in providing ethically appropriate care to women of Pakistani Muslim origin in the UK due to language and cultural barriers. To gain informed consent, midwives should assess whether a woman has a full understanding of the choices available. Culturally insensitive services do not respect the principle of justice. Rather than remaining ‘culturally neutral,’ it is essential to be culturally sensitive to meet their needs. Education and training are required to prepare service providers and midwives for a culturally diverse maternity population.
Megregian 2016 United States^38^	**Aim:** The aim of the study was to examine the current formats, venues, content, barriers, and hours dedicated to ethics education in accredited midwifery education programs in the United States. **Design:** Descriptive cross-sectional a web-based survey.	**Participants:** Program directors of 25 of 49 accredited midwifery education programs. **Context:** The study was conducted across accredited midwifery education programs in the United States.	There is considerable variation in ethics education in terms of content, format, and evaluation among accredited midwifery education programs in the United States. Only 7 (28%) programs offer ethics as a stand-alone class. The majority of programs include the ethical concepts of informed consent, shared decision making, and effective communication. Ethical principles in education: Programs consistently include informed consent, shared decision making, communication, privacy/confidentiality, and professional codes of ethics. Midwifery ethics perspective: The study highlights the potential for developing a distinct midwifery ethic grounded in the principles of woman-centered care, advocacy, and shared decision making.
Megregian et al. 2020 United States^39^	**Aim:** To explore the experiences of U.S. midwives with regard to ethical dilemmas in clinical practice and to identify key sources of their ethics learning. **Design:** A qualitative descriptive thematic study.	**Participants:** 15 Midwives who were or had been in clinical practice and had graduated from an accredited midwifery program in the U.S. **Context:** Midwives, providing clinical care in home and hospital practices, private and academic practices, and independent and collaborative practices.	Midwives described a range of professional ethical dilemmas, including challenges related to negotiating strained interprofessional relationships and protecting or promoting autonomy for women. Midwives in this study reported a range of ethical challenges and minimal classroom education related to ethics. Midwifery educators should consider the purposeful and explicit inclusion of midwifery-specific ethics content in their curricula and in interprofessional ethics education. Reflection and self-awareness of bias were identified as key components of understanding ethical frameworks. As clinical preceptors were identified as a key source of ethics learning, midwifery educators should consider ways to support preceptors in building their skills as role models and ethics educators.
Megregian et al. 2021 United States^11^	**Aim:** To gain consensus regarding key content, competencies, learning outcomes, and teaching strategies for midwifery ethics education. **Design:** An online Delphi study conducted in three rounds.	**Participants:** 11 in Round 1. A total of 41 midwives participated in Round 2, with 12 midwifery program directors, 5 midwifery faculty, 16 preceptor midwives. **Context:** Midwifery education in the United States.	Acquisition of critical thinking skills in ethics decision-making and nurturing of professional attributes such as compassion and courage were identified as essential competencies and learning outcomes, highlighting the dual aims of midwifery ethics education. Shared decision-making was seen as an essential aspect of ethics education across all categories.
Megregian et al. 2021 United States^12^	**Aim:** This study explores midwifery students’ perceptions of ethics education and their opinions about essential components of ethics education. **Design:** A qualitative descriptive thematic study.	**Participants:** 39 students from three midwifery programs in the U.S. **Context:** Graduate midwifery educational programs in the United States.	Thematic analysis identified three primary themes and associated subthemes: 1) current experience and identified needs, 2) the preceptor dilemma, with subthemes the critical role of modeling ethics and powerlessness within interprofessional conflicts, and 3) complicated relationships: advocacy, autonomy and choice. Students. Students relied primarily upon clinical preceptors rather than classroom discussion as a significant source of learning ethics content and ethical behavior. Students called for explicit identification of ethics learning when it occurs, particularly midwifery-specific content, as well as increased opportunities for reflection and integration of their experiences.
Mizuno 2011 Japan^40^	**Aim:** This study describes the clinical and emotional experiences of Japanese midwives working in a large urban general hospital maternity unit, that is, nurses required to assist not only when a baby is born, but also with Termination of pregnancy. **Design:** A qualitative approach using thematic analysis.	**Participants:** 11 midwives who worked in the labor and delivery setting in Tokyo and had over. **Context:** 5 years of work experience in the delivery unit were individually interviewed.	Two major themes emerged: the experience of midwives involved in childbirth and pregnancy termination (three subthemes: confusion about care of the baby and aborted fetus, inability to cater to different mothers’ needs, and establishing emotional control) and professional awareness and attitude as a midwife (three subthemes: consistency with professional principles, suppression of feelings in relation to aborted fetus, and previous and current professional identities). We found that midwives are isolated in this important social moral issue and its accompanying professional confusion. Suppressing their feelings remains the most common way of dealing with the ambivalence of the roles they fulfill. Improved working conditions and enhanced training on aspects of professional ethics would assist in reducing professional confusion.
Newnham and Kirkham 2019 Ireland, United Kingdom^41^	**Aim:** To demonstrate ethics of care as a way of overcoming rhetorical bodily autonomy. **Design:** Discussion paper.	**Context:** Medicalized maternity systems.	The bioethical principle of respect for a person’s bodily autonomy is central to biomedical and healthcare ethics. In this article, its argued that this concept of autonomy is often annulled in the maternity field, due to the maternal two-in-one body (and the obstetric focus on the fetus over the woman) and the history of medical paternalism in Western medicine and obstetrics. The principle of respect for autonomy has therefore become largely rhetorical, yet can hide all manner of unethical practice. We propose that large institutions that prioritize a midwife–institution relationship over a midwife–woman relationship are in themselves unethical and inimical to the midwifery philosophy of care. We suggest that a focus on care ethics has the potential to remedy these problems, by making power relationships visible and by prioritizing the relationship above abstract ethical principles.
Oelhafen and Cignacco 2020 Switzerland^42^	**Aim:** To understand the structure of ethical issues and moral competences in midwifery and to empirically estimate the factors leading to moral distress and its negative consequences. **Design:** Sequential mixed-methods approach with uses a web-based cross-sectional questionnaire survey.	**Participants:** Completed questionnaires=254 Midwives=208 Midwifery students=46 Total= 280 **Context:** 55 worked in outpatient settings. 199 worked in inpatient settings. Almost half the midwives had 10 of more years of work experience.	The core findings of the study are that ethical issues related to asymmetries of power and authority (e.g., lack of respect or inadequate staffing) are the most frequent causes of negative consequences (such as fatigue or job attrition), which serve as indicators of moral distress. Conversely, a positive ethical climate (characterized by respect and interprofessional trust) was identified as the strongest predictor associated with a reduction in these negative consequences. Self-assessed assertiveness also significantly reduced negative consequences. The results emphasize that moral distress in midwifery is less about moral conflicts themselves and more about the frequent experience of constrained situations where the midwife feels a lack of control.
Oelhafen et al. 2017, Finland^43^	**Aim:** To develop effective interventions to increase the ethical competence of midwives in both educational and practical clinical settings. **Design:** Mixed methods exploring perspectives, accessing experiences and generating a model to inform curriculum and policy makers.	**Participants:** 43 semi-structured individual interviews, 6 focus group interviews and survey with 36 participants. Evaluation phase: 100 participants via an online questionnaire. Midwives and Midwifery students. **Context:** 5 partner sites in Finland, Estonia and Switzerland.	The study’s primary finding was a significant gap between theoretical ethical knowledge and clinical reality, as midwifery students often felt a sense of powerlessness when faced with institutional hierarchies and high workloads that conflicted with their idealized views of care. Crucially, the evaluation revealed that although participants showed increased moral sensitivity and awareness, they still lacked the confidence to make autonomous decisions in complex, real-world situations. MidEthics model generated by the data and knowledge gained, based on current literature and a competence framework, is described and discussed.
Oelhafen et al. 2019 Switzerland^44^	**Aim:** To gain insight into the ethical issues midwives come across in their daily work, the key competences and what resources they thought were indispensable to understand and deal with these. On top of this they also wanted to access the phenomena linked to moral distress. **Design:** Phase one of sequential mixed-methods project. Interviews.	**Participants:** 10 health professionals (8 midwives, 1 nurse and 1 senior physician) from Switzerland. **Context:** Health professionals working in in-/outpatient setting, private/public sector, and rural/urban institutions to generate a moral competency profile and describe moral problems and distress during daily work.	External constraints limiting the midwife’s and the patient’s autonomy and resulting interpersonal conflicts were found to be the most relevant ethical issues encountered in clinical practice and were most often associated with moral distress. These conflicts often arise in the context of medical interventions midwives consider as not appropriate and situations in which less experienced midwives in particular observe a lack of both interprofessional communication and trust in their professional competence. Ethical issues related to late abortions or pre-natal diagnostics and selective abortions were also frequently addressed, but many midwives involved had learned to cope with them.
Pezaro et al. 2025 United Kingdom^45^	**Aim:** To advocate for gender-inclusive language in midwifery and perinatal services and linking it to an ethical imperative and a requirement for reproductive justice. **Design:** Qualitative and discursive by using philosophical and sociological analysis to justify a set of practical professional recommendations.	**Context:** 13 authors who included individuals who identify as trans, queer, gender-diverse and cisgender. They also were midwives, midwifery students and academics in midwifery, sociology and philosophy.	The authors argued that evolving midwifery language is not just a social adjustment but a professional responsibility necessary to maintain public trust and meet established ethical standards of practice. This is shown by discussions on how ‘sexed language’ causes clinical hard, violating the bioethical principle of non-maleficence. The principal of justice requires equal healthcare access for all birthing people therefore inclusive communication is a professional duty to upload reproductive justice. Midwives should move beyond tradition bioethics towards care ethics or queer bioethics to properly respect the bodily autonomy and individualized needs of a diverse population.
Ramsayer and Fleming 2020 United Kingdom^46^	**Aim:** To discuss some of the key ethical and legal concepts that are relevant to midwives’ roles in the provision of abortion services. **Design:** Discussion. Philosophical integration paper.	**Context:** Conscience and conscientious objection: The midwife’s role in abortion services.	This article shows that the decision to provide or object to abortion services remains ethically very complex because arguments exist both for and against its provision. Being with women can be interpreted differently and individual situations of care are multifaceted. Conscientious objection to abortion services is a highly contentious issue that has an overall importance to midwives. Noting that decisions are individual, may change or may be situationally dependent; a definitive position of midwives for or against conscientious objection cannot be assumed. Midwifery ethics center on the philosophy of ‘being with women’, the protection of moral integrity through conscientious objection and the tensions between professional duties and personal values.
Rost et al. 2025 Switzerland^47^	**Aim:** To identify, comprehensively map, and categorize possible causes of moral distress among midwives, and to identify knowledge gaps. **Design:** Scoping review conducted following Arksey and O’Malley framework and PRISMA-ScR guidelines.	**Participants:** Included 43 empirical studies (69.8% qualitative, 18.6% quantitative, 11.6% mixed methods) published in English, German, French, or Italian. **Context:** Studies represented midwives, midwifery students, and nurse-midwives from 22 countries (mostly high-income: Europe, Australia/Oceania).	Moral distress among midwives arises from conflicts between external constraints and personal moral standards, reflecting limited moral agency. Eight clusters of causes were identified: 1) Societal disregard, 2) Contemporary birth culture, 3) Lack of resources, ,4) Institutional characteristics, 5) Interprofessional hierarchies, 6) Interpersonal mistreatment of service users, 7) Defensive practice, and 8) Challenging care situations. These conditions “undermine professional autonomy, dehumanize care, and erode midwives’ moral integrity.” (p.1394) The authors note that “midwives’ autonomy is being eroded by restrictive institutional and regulatory systems… which reflect insufficient legal and policy protection for midwifery practice across contexts.” (p.1396). Across these contexts, midwives faced hierarchical, medicalized, and resource-limited birth cultures, often shaped by institutional policies, gendered norms, and insufficient legal protection of midwifery autonomy.
Schorn 2007 United States^49^	**Aim:** To determine midwives’ practices and beliefs about unilaterally discharging clients from their practice, and to identify reasons for discharge or for not discharging a client. **Design:** Quantitative descriptive study using self-administered questionnaire.	**Participants:** 111 Certified Nurse-Midwives (CNMs) across the U.S. **Context:** Survey included 14 items assessing frequency, reasons, and attitudes regarding client discharge.	U.S. midwifery practice where CNMs operate within collaborative models, legal frameworks related to abandonment, and organizational policies that mandate ethical, legal, and documentation standards. In addition to the legal issues, midwives need to consider the ethical principles of patient autonomy, beneficence, nonmaleficence, justice, and veracity in situations. A clinician’s fiduciary duty can be particularly challenging when ethical principles are in conflict.
Simbar et al. 2023 Iran^48^	**Aim:** To develop and validate the Iranian Code of Ethics for Midwives (ICEM) to improve quality of care and guide ethical midwifery practice. **Design:** Mixed-method sequential study.	**Participants:** Interviews with 14 experts (midwives, ethics professionals, educators, and managers). **Context:** Review of national and international midwifery codes of ethics; Validity assessment by 15 experts using Content Validity Ratio (CVR)and Content Validity Index (CVI).	207 codes were extracted from a content analysis which were categorized into 23 sub-categories and 6 main categories. The extracted codes were considered as the items for ICEM that were completed by a review of the selected national and international code of ethics for Midwives. The content validity and ratio assessment of the items demonstrated an average CVI = 0.92 and CVR = 0.85. Then, the final version of ICEM was developed with 92 items in 6 domains about; 1) 'professional commitments' with 30 items; 2) 'providing midwifery services to the client and her companions' with 26 items; 3) ‘relationship with colleagues’ with 11 items; 4) ‘herself' with 6 items; 5) ‘education and research’ with 8 items; and 6) ‘management’ with 11 items.
Thachuk, 2007 Canada^50^	**Aim:** To examine how the Canadian midwifery model of care aligns with feminist relational autonomy theories, and how informed choice in midwifery enhances reproductive autonomy. **Design:** Conceptual / Theoretical paper (no participants). Uses feminist bioethics, critiques of medical informed consent, philosophical argumentation, and analysis of midwifery regulatory policy.	**Context:** Canadian midwifery transitioning from a social movement to a regulated profession. Increasing medicalization, regulatory pressures, documentation requirements, and the tension between client choice vs professional protocols. The article evaluates the philosophical foundations of informed choice within Canadian midwifery.	Canadian midwifery’s emphasis on empowerment and informed choice offers an exemplary standard of practice that maximizes women’s reproductive autonomy. Regulation has increased occupational autonomy on a collective level, but may have decreased the autonomy of the individual midwife. The needs and wishes of the client may become secondary to the protocol and policies to which midwives are currently bound. Informed consent retains more of its paternalistic predecessor than its adherents would wish to admit. Informed choice is more reflective of relational autonomy, emphasizing social situatedness, power dynamics, and shared responsibility. Continuity of care enables trust, deeper disclosure, and more authentic autonomous decision-making.
Thompson 2004 Australia and United Kingdom^51^	**Aim:** To presents some of the tools available to professionals who take up the challenge of using ethical thinking to shape best practice in any setting. **Design:** Discussion paper about teaching ethics.	**Context:** The midwife’s role as teacher of ethical reflection, whether formal or informal Midwives face ethical issues of greater or lesser significance throughout their careers.	Key ethical concepts in childbirth are: Choice, control, autonomy. Fetus as ‘silent’ client – best interests may conflict with that of the mother. To teach ethics, midwives need to: Know themselves, know their students, know their subject, know their clients. Ethics best taught by ‘doing’ ( rather than theory). Use of codes (e.g. ICM) can be useful. Examples of such structural constraints include, Lack of access to reproductive health care Injustice in the way in which services and benefits are administered Discrimination against women and girls at all levels: economic, cultural, and educational
Thompson 2003 Australia^52^	**Aim:** Consider the lived experience of mothers and midwives, and their ethicality of their reality. **Design:** Narrative enquiry (feminist) interview study.	**Participants:** 8 midwives, 8 childbearing women. **Setting:** Queensland, Australia.	The central theme to emerge from these narratives was the use and abuse of power in relationships. The following major themes, ‘values conflict’, and subthemes of ‘workplace/service provider versus personal/professional midwifery ethics’ and ‘not valuing individuals’. Traditional bioethics, with its universal principles, abstract theory and objective problem solving in dilemmas, does not resonate with the ethics identified by these mothers and midwives, that is, the ethics of engagement. This latter ethics, which until now has been implicit in midwifery within the shared traditions and real-life experience of childbearing women and midwives, has been made explicit by listening to their voice in this narrative enquiry.
Thompson 2002 Australia and United Kingdom^53^	**Aim:** To demonstrate abstract principles are inadequate for midwifery practice. **Design:** Discussion paper related to feminist relational ethics.	**Context:** Australian and UK codes of ethics for midwives reveals the conflict between workplace and personal/professional ethics, and demonstrates some of the difficulties with using existing codes of ethics to guide practice.	Normative ethical theory and abstract universal principles, on which most of the existing professional codes are based, are inadequate for well-woman centered midwifery practice. They focus on right and wrong ‘action’ rather than ethical engagement between people, and do not inform the practitioner how to transform principle into practice. Midwifery values, which are both implicit and explicit in narrative and literature, indicate that the mother–practitioner relationship and knowing the person (mother and midwife knowing each other; continuity of care and carer) are of prime importance.
Thompson 2004 United States^54^	**Aim:** To present a rights base for midwifery care of women and childbearing families. **Design:** Discussion paper.	**Context:** The influence of values on how women are viewed within cultures and societies, universal ethical principles applicable to health care services, and human rights based on the view of women as persons rather than as objects or chattel.	Focuses on the interface of values, ethics, and human rights in providing midwifery care to young girls and women throughout their lives. This interface constitutes a human rights framework for midwifery care that respects all persons, promotes informed decision making, takes measures to prevent any acts of violence or discrimination against clients, maintains privacy, and promotes safety for all seeking midwifery care. A human rights framework for midwifery care—a model supported by codes of ethics, the midwifery philosophy of care, and standards of practice.
Tömmel 2024 Germany^55^	**Aim:** To get from a phenomenology of birth towards an ethics of obstetric care. **Design:** Feminist phenomenology narrative.	**Participants:** Birth accounts of birth experiences (9) & birth narratives from interview studies (5).	Feminist care ethics most important philosophical source of relational understanding of autonomy. Author CONFLATES obstetric and midwifery care as if the same informed consent process alone cannot do justice to ethical claims of women in the course of reproductive decisions - a relational understanding of autonomy is essential for the ethics of obstetrics and midwifery obstetric violence occurs when relationality in birth is violated.
Tuming and Zolkefli 2024 Bruinei^56^	**Aim:** The views of midwifery students regarding ethics education were examined in this study. **Design:** Qualitative descriptive research design.	**Participants:** Six final-year midwifery students who had completed clinical placements were emailed open-ended interview questions. **Context:** Midwifery students.	Many midwifery students feel unequipped with skills necessary to recognize and resolve ethical dilemmas – this then impacts midwifery workforce ethical expertise. Two broad themes identified: (1) Adequacy in knowledge and (2) Between expectation and reality. Students have reported an ethics course in the midwifery curriculum helped improve knowledge of ethical principles and increased awareness of ethical considerations in clinical environments connection between students’ ethical education & experience with ethical concerns in the clinical setting.
van der Waal and van Nistelrooij 2022 the Netherlands^57^	**Aim:** Demonstrate identification of the structural form of violence that a care ethical relational approach to reproductive care is up against: that of “maternal separation”. **Design:** Discussion paper.	**Context:** Confronted with reproductive and obstetric violence globally, we show that a hegemonic racialized, instrumentalized, and individualized conception of pregnancy is responsible for a severance of relationalities that are essential to safe reproductive care.	The emphasis on the maternal–fetal conflict in ethics, obstetric practice, but also in popular culture, as the ethical dilemma and medical problematic of pregnancy, not only unjustifiably neglects other issues but also reproduces the severance of the relation between mother and child. Instead of trying to understand the relationality of the reproductive subject and the event of childbirth. Suggest relational ethics or praxis regarding abortion, pregnancy, and childbirth care through a re-imagination of the reproductive, maternal, and midwifery relationalities that can challenge and interrupt individualized subjectivity.
van der Waal 2025 the Netherlands^58^	**Aim:** To demonstrate the primary way of struggle against obstetric violence of the mothers, midwives, midwives in training, and doulas in this study, is through a praxis of an abolitionist ethic of care. **Design:** Responsive evaluation is a democratic and dialogical method, offering room for interaction and exchange of experiences and ideas among participants and researchers.	**Participants:** 31 participants were recruited by the first author of this paper; ten mothers, eleven midwives, five doulas and five midwives in training. **Context:** Dutch maternity care.	The most listed occurrences of violence in obstetrics in this study are: (1) obstetric racism; (2) epistemic injustice, mainly playing the dead baby card; (3) physical violence, consisting of interventions without consent; (4) penetrative violence, that is, violence linked to, or reminiscent of, rape or sexual assault; and (5) unconsented and/or unwarranted, and/or unwanted vaginal examinations. In the quote from a midwife in training below, all five of these forms of violence are present in a situation wherein a vaginal examination is being done.
Vincifori 2014 Italy^59^	**Aim:** To describe the professional identity of Italian midwives as depicted by the Italian Midwives Ethical Code and expressed in everyday work. **Design:** Survey – Cross-sectional study.	**Participants:** The instrument was delivered to 235 midwives of the districts of Como, Lecco, and Sondrio (Lombardia, Italy). **Context:** Midwives working in hospitals, or in communities or as independent midwives.	In the health field, the value system of the individual is linked to the membership culture and both are included in the codes of professional ethics. The most widespread profile emerging from the survey results seems to be the so-called ‘hybrid midwife’, who experiences the contradiction between what can actually be achieved in a hospital environment and the core values of midwifery.
Yanow 2021 United States^60^	**Aim:** Discussion paper about the role of midwife in self-managed abortion. **Design:** Discussion paper.	**Context:** In the United States, although the practice of self-managed abortion is criminalized in many states, people are finding abortion pills in a range of ways. Some people are obtaining misoprostol prescriptions for ulcers or arthritis it is available from veterinary supply stores. Combination packs of misoprostol together are available on the internet through a range of sources.	Codes are grounded in bioethical principles. Nonmaleficence, which refers to doing no harm, is an ethical principle requires that the midwife avoid harming the patient, which can be derived from action, lack of action, or lack of knowledge. Midwives have an ethical obligation to become knowledgeable about options counselling. Without gaining and sharing this knowledge, midwives may place their patients at risk of unnecessary harm and, in doing so, violate their ethical obligations to their patients. This ethical obligation; it requires action on the part of the midwife to benefit the patient. In the context of bioethics, justice refers to the need for health care to be fair in its provision. Patient education and support can reduce patient risk, and as such represents a harm-reduction strategy. Within midwifery ethics to promote access to health care and end disparities to make information about abortion pills and how they are used more widely available, and for midwives to participate in that information-sharing.
Zolala 2019 Iran^61^	**Aim:** To determine the severity and frequency of moral distress in midwives working in birth centers. **Research design:** This study is descriptive cross-sectional research. Researcher-made questionnaire was used to gather data.	**Participants and research context:** A total of 180 nursing midwives working in the Labor ward of the public birth centers (maternity ward) affiliated to Shahid Beheshti University of Medical Sciences were included to the study by census.	The highest severity and the lowest frequency of moral distress were obtained for the assistance for abortion, and the lowest severity of moral distress was related to the organizational domain. However, the highest frequency of moral distress was related to futile care field. The mean of moral distress severity in the midwives with associate degree was significantly lower than other levels of education. Also, there was a significant relationship between age and moral distress frequency (p ¼ 0.010).

### Extraction stages

Each author was allocated five articles from which to extract the data using the table headings. Each reviewer was familiar with the review question and the data to be extracted. Data extraction was conducted in accordance with JBI scoping review methodology, with data extracted by a primary reviewer checked by the primary author, and discrepancies reviewed through team verification and consensus processes^13,14^.

Data extraction for this scoping review was conducted as an iterative and interactive mapping process^13^, and we followed the basic content analysis approach recommended for scoping reviews^13-15^. The process included: 1) Preparation: becoming familiar with the data, organizing the material for analysis, and preparing tables for coding; and 2) Organizing: the researchers coded the data, this is where classification and reduction of the data occurred; and 3) Reporting: summarizing and presenting the findings in a clear, structured table and summarizing narrative. An open-coding approach was applied to allocate concepts or characteristics into overarching categories. This process involved assigning descriptive codes to capture key ethical concepts and grouping these codes into broader categories to map patterns across the literature. These categories were then summarized narratively to provide a descriptive overview of the evidence (Supplementary file Table 1).

### Data analysis and presentation

The evidence presented responded directly to the review objective and research question. Data were displayed in tabular form to demonstrate the characteristics of each article and to map ethical topics and concepts across the included studies^13^. This approach aligned with the descriptive purpose of scoping reviews, which aim to map the breadth and nature of the available evidence. During data extraction, concepts were coded^16^ so that key ethical issues brought forward in each article could be keyword and conceptually mapped (Supplementary file Table 1). The process centered on identifying key characteristics and factors relevant to the review question.

### Extraction reliability and validation

Critical appraisal was not a requirement^14^. However, it is worth noting that all authors were involved in extraction and resolved questions of quality through regular meetings.

### Data management and sharing

The tables were uploaded on SharePoint, and all authors could access the tables and be involved in the verification process through meetings.

## RESULTS

Fifty articles were included in the review^6,8,10-12,17-61^. The characteristics of the articles are given in Table 2 and include details such as author, year, country, research aim and design, participants, context, and main findings related to midwifery ethics. Figure 2 provides graphical representation of the geographical region, temporal distribution, study design and care context of the sourced literature.

**Figure 2:**
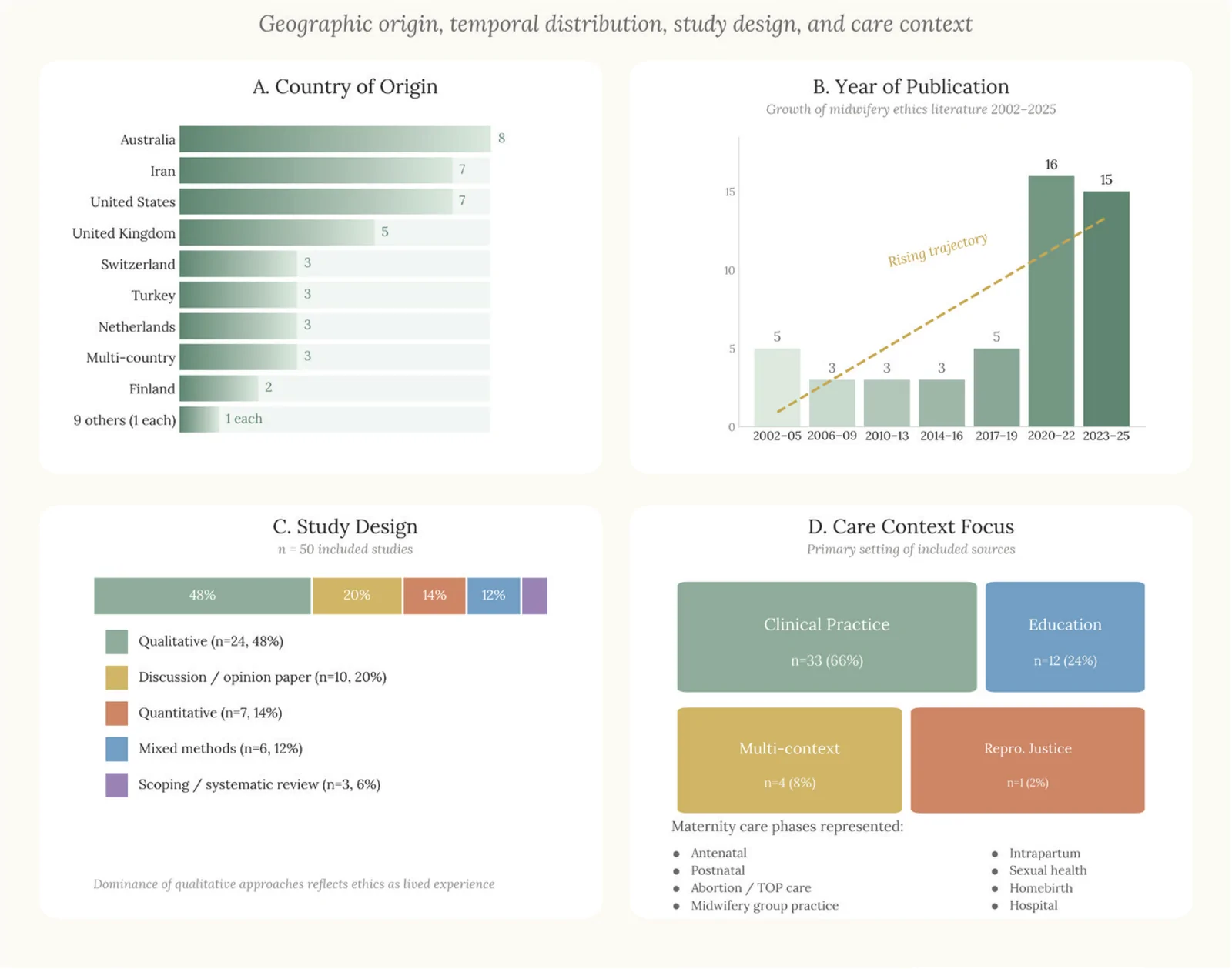
Schematic representation of the characteristics of sourced literature including geographical origin, temporal distribution, study design and care context of midwifery ethics (Articles retained n=50 from MEDLINE, MIDIRS, CINAH, PsycINFO and Emcare, 2002–2025)

Figure 3 demonstrates the frequency with which ethical concepts are discussed (reach) but also how centrally they are examined (depth). Four distinct patterns emerge. Core concepts such as relationship, autonomy and power which are both widespread and the focus of the studies. These represent the well-established or foundational aspects of contemporary midwifery ethics. Next, ambient concepts, including midwifery philosophy, woman-centeredness are commonly referenced but usually serve as background framing rather than the core topic. Concentrated topics, in particular care ethics, obstetric violence and informed choice appear in fewer studies but are examined in substantial depth. Far from being marginal, they should not be read as neglect but as specialist or emerging foci. Finally, peripheral concepts such as moral distress, reproductive justice and advocacy are less frequently discussed and rarely central. Indicating a more consequential gap both understudied and under-developed. These findings together highlight that prevalence alone can obscure important patterns, but as Figure 3 highlights, can be analyzed to reveal areas of established consensus, emerging attention and potential gaps, particularly in relation to reproductive justice.

**Figure 3:**
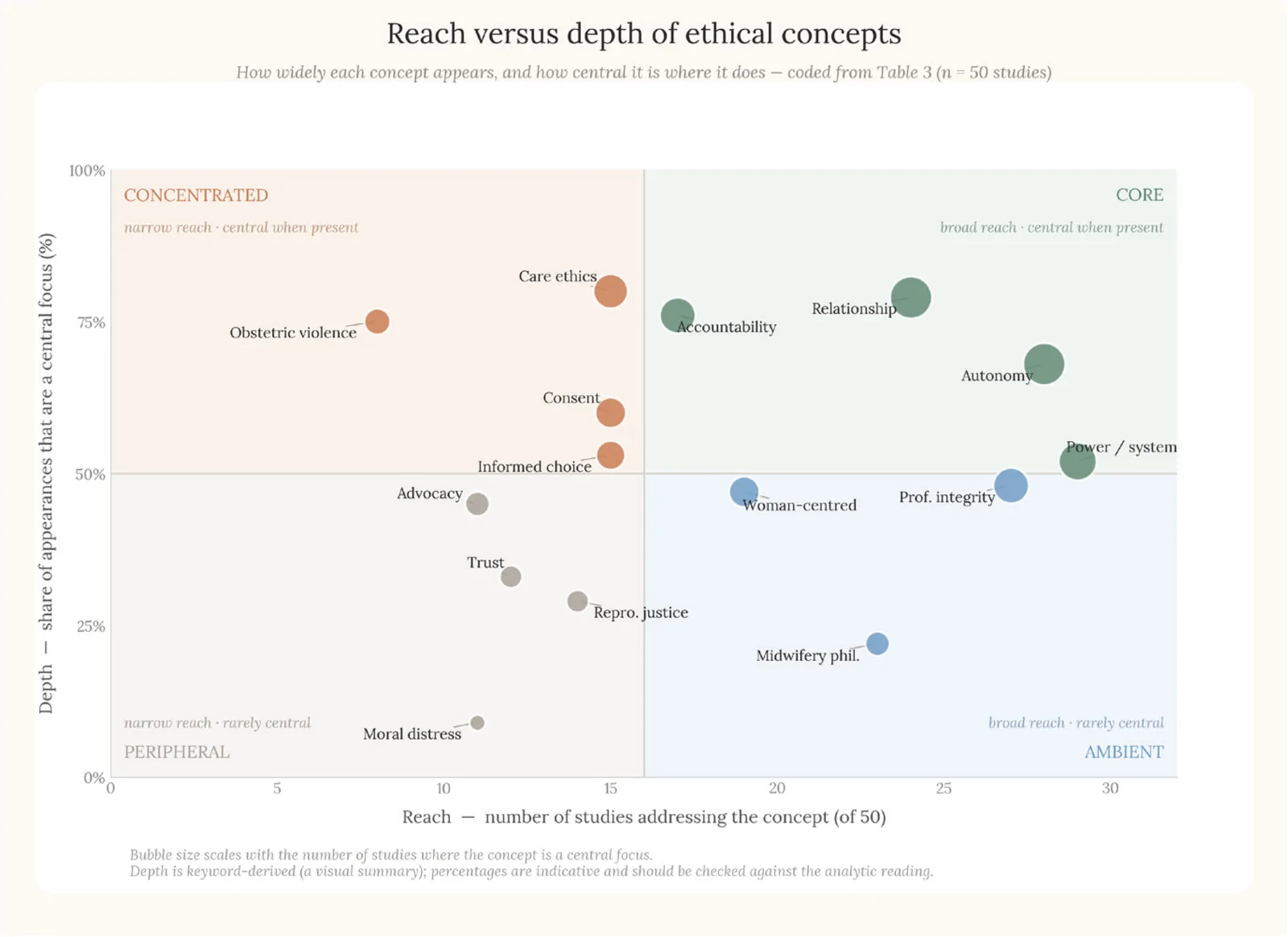
Schematic representation of reach versus depth of ethical concepts across the 50 included studies, showing four distinct patterns; core concepts that are both widespread and central (relationship, autonomy), ambient concepts that are common but rarely central (midwifery philosophy), concentrated concepts that are less prevalent but examined in depth (obstetric violence); and peripheral concepts that are infrequent but emerging (moral distress, reproductive justice)

### Narrative data synthesis of ethical concepts

Across the literature, four interrelated ethical topics were identified as foundational to ethical midwifery practice. These were synthesized and mapped in Figure 4, a four domain framework for midwifery ethics, which is derived from convergent open-coding across the included literature. The diagram organizes the coded ethical concepts into four thematic domains and communicates their interconnected, dynamic relationship of ethical concepts. The figure is next further explained narratively.

**Figure 4:**
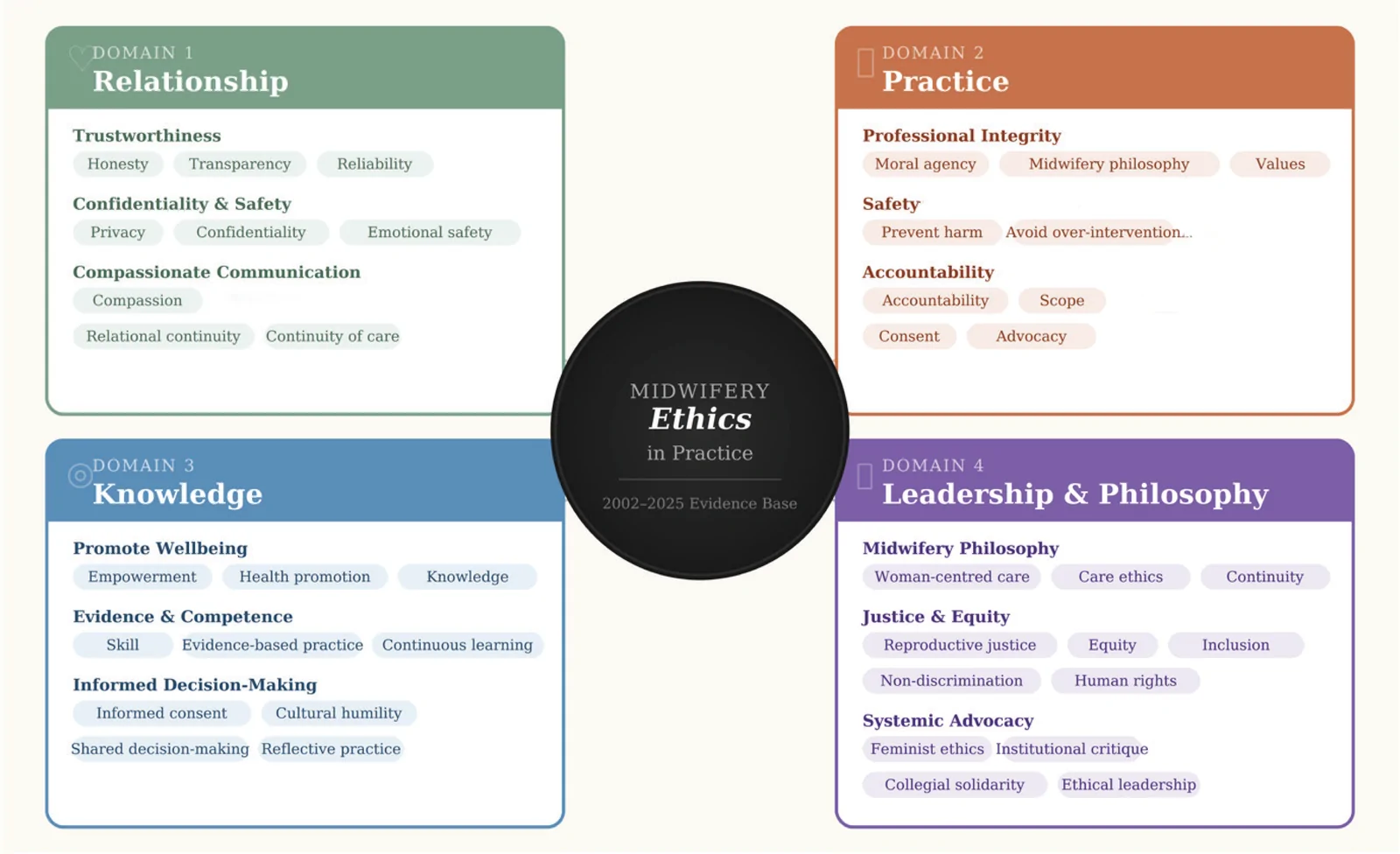
Schematic representation of findings to four domains that capture midwifery ethics from the literature sourced 2002–2025. The four domains include relational ethics, ethical practice, ethical knowledge use, and ethical leadership

*Relational ethics*

Relational ethics centers midwifery practice. The results describe the importance of the midwife-woman relationship and subsequent care that is grounded in understanding the woman’s values and context. Midwifery ethics were best actualized in relational continuity and attentiveness, regardless of model of care. Key codes included trust, confidentiality, secure environments, discretion, compassion, therapeutic communication, and emotional safety.

*Practice of ethics*

The practice of midwifery ethics was rooted in including alignment with midwifery values and midwifery practice. The practice incorporates the bioethical principle of non-maleficence through preventing harm, avoiding unnecessary intervention and prioritizing safety. Codes related to responsibilities relating to midwifery philosophy, scope of practice, duty to act, reporting, advocacy and accountability.

*Ethical use of knowledge*

Ethical knowledge use involves promoting wellbeing and empowerment through evidencebased practice, and sharing knowledge, which equalizes power. Continuous learning and reflection were essential for ethical practice. Codes included evidencebased practice, sharing knowledge, continuous learning and reflective practice.

*Ethical leadership*

Ethical leadership is grounded in midwifery philosophy which centers on womancentered relational care, physiological birth, continuity of care, and advocating for the full scope of midwifery practice. It also encompasses justice, equality, equity, inclusion, nondiscrimination and fair access to resources. Codes included reproductive justice, equity, nondiscrimination and advocacy.

## DISCUSSION

Across the fifty articles included in this review, midwifery ethics emerges as a geographically disperse yet conceptually coherent field. The literature is mainly qualitative inquiry reflecting midwifery’s emphasis on lived experience, with some discussion papers addressing ethical theory and thinking in midwifery. This scoping review identified that midwifery ethics is increasingly attentive to the moral complexity of midwifery work and grounded in relational woman-centered and justice-oriented care, which aligns with midwifery philosophy.

There have been continuing efforts globally to realize the importance and impacts of midwifery care as seen in the State of the World Midwifery Reports^62^, the Lancet Series in Midwifery^63^, and the World Health Organization’s recent call for global expansion of midwifery model of care^64^. Midwifery plays a critical role in achieving the Sustainable Development Goals by improving access to maternal and newborn health, reducing inequities and strengthening health systems^62-64^. Alongside this work, scholars have been highlighting the unique component of the success of midwifery care: the centrality of relationship^65-67^.

Addressing relationality in midwifery care has more recently caused a turn towards ethics, specifically, care ethics^8,41^, primarily due to the identification that the relationship is disrupted by fragmented, medicalized, institutional models of care^57,68^. The reliance on bioethics and the fallibility of principle of autonomy have been problematized due to their failure in safeguarding ethical care^57,58^, as evidenced by extremely high reports of disrespect, abuse and obstetric violence experienced by women and birthing people across the world^9,68^. Midwifery philosophy has been mapped against care ethics theory, which was found to provide a more aligned framework for the kind of relational care that midwifery prioritizes^8,17,69,70^. Additionally, midwifery’s position as the only maternity care profession with a history of ancient practice and embodied childbirth support has been identified as providing a valuable, singular expertise – termed relational midwifery thinking^71^. Further theoretical work contends that attentiveness, which sits at the heart of relational practice, enables a ‘loving praxis’ that is essential to uphold the right to bodily self-determination and therefore necessary for justice^69,70^.

Recent international work further reinforces that ethical midwifery practice is grounded in the profession’s core philosophical commitments. An international Delphi study by Buchanan et al.^6^ identified globally agreed ethical features that closely align with the domains of relationship, practice, and knowledge, highlighting their centrality to ethical midwifery. Relational ethics emerged as foundational, with continuity identified as essential to ethical partnerships between midwives and women. These elements support emotional safety, and respectful care, with features long recognized as integral to the midwife–woman relationship^66,67,69,72^ and consistent with the ICM’s emphasis on dignity, respect, and nonauthoritarian care^5^.

The ICM Philosophy and Model of Midwifery Care establishes that ethical midwifery is fundamentally relational, rights-based, culturally grounded and emancipatory^3,5,6,8^. When viewed through this lens, existing literature reveals a persistent gap between midwifery’s ethical foundations and the realities of the contemporary maternity systems^6,9,35,68^. These authors describe how institutional constraints, risk-adverse cultures or fragmented models of care limit midwives’ ability to practice in ways that align with their midwifery philosophy. These findings suggest that ethical challenges are more often produced by systems rather than midwives themselves. Studies documenting moral distress among midwives^23,24,36,41^, reveal ethical tensions arising from resource constraints, restrictive and hierarchical cultures, and institutional pressures of increasingly unsustainable health systems.

This review describes a midwifery ethic that coheres around four interconnected domains: Relational ethics, the Practice of ethics, the Ethical use of knowledge, and Ethical leadership. These articulate professional ethics, specific and unique to midwifery. Relational ethics emphasizes continuity, and respectful partnerships; the practice of ethics draws on care ethics attributes; the ethical use of knowledge underscores evidence-based practice and shared knowledge to equalize power imbalances; and ethical leadership that extends midwifery philosophy and commitment to advocacy, equity and reproductive justice. Together these findings provide a clear evidence-informed framework to inform the 2025-2026 revision of the ICM International Code of Ethics for Midwives, whilst remaining responsive to ethical realities in global maternity care.

### Limitations

This scoping review has several limitations. First, consistent with JBI methodology, the review aimed to map the breadth of literature rather than appraise study quality. Second, the search strategy was limited to English language publications and selected data bases, which may have excluded relevant work, and increases the risk of publication bias Finally, because the field of midwifery ethics is evolving, the findings represent a snapshot in time and may require updating as new evidence emerges.

## CONCLUSIONS

This scoping review brings together ethical concepts specific to midwifery care, practice and education. Midwifery ethics is evolving, with relational care ethics and reproductive justice orientated ethics as central to understanding contemporary midwifery ethics. The findings offer an evidence-informed foundation to support the development of a renewed, globally relevant International Code of Ethics for midwives.

## Supplementary Material



## Data Availability

The data supporting this research are available from the authors on reasonable request.
