# Supplementary material for: Midwifery ethics in care, practice, and education: A scoping review
Source: Eur J Midwifery. 2026 Oct 9;10:10.18332/ejm/231691. doi: 10.18332/ejm/231691 (PMC13653359; doi:10.18332/ejm/231691)
Supplement: Supplementary file 1 [file EJM-10-45-s1.pdf]

EUROPEAN JOURNAL
OF MIDWIFERY

Supplementary file

© 2026 Buchanan K. et al.

DOI:
10.18332/ejm/231691

The content has been provided by the author(s) and has not been reviewed, verified, or endorsed by European Publishing. It may not have undergone peer review. The views, opinions, and recommendations expressed are solely those of the author(s) and do not necessarily reflect the position of European Publishing. European Publishing accepts no responsibility or liability for any consequences arising from the use of, or reliance on, this content.

Table 1. Data extraction table

	Author year	Key ethical concepts addressed in the paper	Coding to key words/ concepts	Relates to Statement in the Code
1	**Berkiten Ergin, 2013**	Professional competence, professional knowledge and skills, trustworthiness, respect for privacy, empathy, responsibility, justice and equality, professional integrity, and maximum benefit with minimum harm<br>Midwives emphasized respecting privacy, empathy, equality, avoiding discrimination, and achieving maximum benefit with minimum harm.	professional competence,<br>trustworthiness,<br>respect for privacy, empathy,<br>responsibility,<br>justice and equality, professional integrity,<br>beneficence<br>minimum harm	**Domain 1 b.**<br>**Domain 3 g.**
2	**Buchanan, K. et al 2025[a]**	Philosophical foundation of midwifery ethics; Relational and trust-based care; Embodied ethics; human rights, reproductive justice; Advocacy and reflexivity.<br>Professional competence and accountability<br>Woman-centred autonomy; Inclusivity and cultural respect. care that aligns with the woman's values and context.<br>Resistance to institutional and patriarchal constraints.<br>Reflexive practice; Ethical self-awareness.<br>Ethical midwifery requires compassion, inclusivity, and respect for cultural, spiritual, and social differences.	Midwifery philosophy<br>Relational and trust-based care;<br>Continuity<br>Respectful care, compassion<br>Human rights Reproductive justice<br>Advocacy solidarity<br>Informed consent<br>competence accountability.<br>Woman-centred autonomy<br>Holistic Inclusivity and cultural respect.<br>empowerment;<br>patriarchal constraints.<br>Reflexive practice; Ethical self-awareness.	**Domains 1 -4 2c**
3	**Buchanan, K et al., 2025[b]**	Relational partnership' with woman foregrounds mutual respect, trust, and shared decision-making<br>Relationship with woman fosters ethical awareness<br>'Learning to advocate'.<br>Midwifery students identified ethical issues during practical placement related to consent, overuse of interventions, insensitive practice, and overuse of technology, but felt powerless to act, which caused subsequent moral distress.	Relationship<br>Adv Obstetric violence<br>System conflict<br>Woman-centred<br>Birth philosophy<br>Reproductive justice advocacy<br>Informed choice<br>Consent<br>Care ethics	**1a**<br>**2d**<br>**4c**<br>**4a**<br>**2d**<br>**4c**<br>**1c**<br>**3b**<br>**1c**

Table 1. Data extraction table

		Ethical care ensures choices made by the woman are freely understood and consented, particularly given that the woman has long term responsibility for the consequences of those decisions.<br>Care ethics is visible in this layer, where midwifery care as seen as caring in and off itself as ethical.<br>The woman is centred, and her ethical values and context are primary<br>Additionally, the hierarchical and oppressive health care structures can inhibit midwives from advocating freely for themselves, their profession or for women in their care	Autonomy<br>Midwifery philosophy	**4a**
4	**Buchanan, K et al., 2023**	Individuals' values and context' placed the woman at the centre of the care, in determining what is ethical.<br>prioritize relational, individualized care to counter medicalization and disrespect,<br>integrating feminist care ethics theory to enhance everyday practice and reduce harm in maternity systems.<br>A conceptual model Woman-centred ethics<br>is offered to enhance everyday ethical midwifery care.<br>Woman-centred ethics involved midwifery solidarity (strong relationships), transparent knowledge (unbiased information), and physiology supported (agency/choice), leading to claiming power (embodiment, empowerment).	Woman-centred<br>Individual values/context<br>Continuity Relationships,<br>Feminist ethics<br>Woman-centred ethics<br>Transparent information<br>Autonomy (agency)<br>Empowerment	**3 b**<br>**1b**<br>**3b**<br>**1a**
5	**Buchanan, K et al., 2022[a]**	Care ethics categories are: relationship, caring practices, context and attention to power, that together offer a framework for enhancing ethical sensitivity for practice<br>Relationships are central, facilitating ethical care through mutual understanding.<br>understanding the person at the centre of the care, providing competent care<br>Care ethics addresses maternity ethical challenges, human rights	relationship,<br>caring practices,<br>individual context<br>attention to power,<br>competence<br>individuals' values<br>human rights	**1b**<br>**1d**<br>**2a**
6	**Buchanan, K., 2022[b]**	Continuity of care, particularly with same caregiver, essential to ethical care whereby individualized care relationships are essential for women	Relationship, continuity<br>Individualised care	**Domain 1a, b, c**<br>**1 a.**

		to actualize autonomy and choice, levels power and that the socio-cultural contexts of birth are significant.<br>Ethical midwifery focuses on promoting physiologic birth and trust in the inherent processes of labor/birth same philosophy of birth,<br>Ethical care was demonstrated when they women had the power to make decisions and to exercise agency	Transparent information<br>Power to make decisions, autonomy<br>Physiologic birth<br>Sociocultural context, Significance of birth<br>Strengthen woman capabilities<br>Shared birth philosophy<br>Awareness of power	**3b**<br>**3c**<br>**4d**<br>**1a**<br>**1d**
7	**Carvajal et al., 2024**	Midwives' identity is woman-centred, with high value placed on their role protecting life. These two aspects of midwives' identity are in contradiction when providing abortion care. Midwives' identity results from and informs midwives'<br>Midwifery identity is shaped by core values but are interpreted individually, resolving tension between required practice and personal core values. The value of "woman-centred" takes on different meanings in practice.<br>Midwifery identity begins during education / training, which requires midwifery educational institutions to create, define, guide and train students in the core values and practices of midwifery, developing that identity. This variability in midwives' response to the abortion law seems to relate to how strongly they adhere to being 'woman-centred'	Woman-centred<br>Philosophy<br>Respect for choice<br>Autonomous<br>Midwifery knowledge<br>Reproductive justice<br>Midwifery philosophy<br>Scope of practice<br>Core values<br>Professional regulation<br>Professional identity<br>Ethics education	**Domain 1c**<br>**Domain 1b**<br>**2d**<br>**4a**<br>**4d**<br>**2b**
8	**Ergin et al., 2013**	The five professional values that the participants ranked as most important were, in order, (a) caring for mother-child health, (b) being responsible, (c) having professional competence, (d) having respect for privacy and (e) being reliable. avoiding conflicts of interest, protecting privacy, avoiding deception, reporting faulty practices, seeing the mother and newborn as separate beings and preventing harm. suggested additions made by the participating midwives to the ethical code list included (a) being open to professional developments and following them, (b) giving priority to professional ethics and protecting them, (c) being disciplined in professional practice, (d) being aware of her powers and limitations, (e) valuing patients and letting them know that they are valued and (f) being able to warn her colleagues if something goes wrong.	Caring practices<br>Responsibility<br>Competence accountability<br>Respect for privacy<br>Avoid deception<br>Conflict resolution privacy,<br>Trust<br>Reporting<br>Dyad<br>Prevent harm<br>Continuous education<br>Prioritise code and protect it<br>Awareness of power	**Domain 2a, g**<br>**Preamble to relationship s**<br>**2 d i ii**

			Collegial support<br>Letting women know their valued	
9	**Firoozehchian et al., 2022**	Integration of science and practice in midwifery; 1-2 compliance with ethics and regulations in providing midwifery care; 1-3 Improving the independent function of midwifery; 1-4 evidence-based practice<br>Category 2: Holistic Midwifery Care; 2-1 involving women and their families in providing midwifery and reproductive and sexual health services; 2-2 providing education and support to women and their families for promoting reproductive and sexual health<br>Category 3: Effective interaction; 3-1 using communication skills to provide midwifery and reproductive and sexual health services; 3-2 effective and coordinated communication with other people providing midwifery and reproductive and sexual health<br>Category 4: Personal and professional development; 4-1 Feeling responsible and committed to learning for yourself and your peers 4-2 accountability in providing midwifery and reproductive and sexual health services	Midwifery knowledge<br>Compliance with regulations<br>Evidenced based practice<br>Holistic care including families<br>Providing education<br>Communication skills<br>Accountability for learning	**Domain 3f**<br>**Domain 3b**<br>**Domain 1e**<br>**Domain 2a**
10	**Fontein-Kuipers et al., 2018**	Three themes emerged: (1) *Loyalty* - the conflict between wanting to be loyal to the woman's wishes and expectations AND to guidelines, scientific evidence and to the collaborative relationships with other professionals – the value of women's childbirth experiences *versus* the value of good health outcomes (influenced by the midwife's risk perception, the healthcare system and organization of care). (2) **Responsibility** - the conflict between respecting the woman AND doing her justice as a person and the social norm in maternity services - women's autonomy and individuality *versus* the midwife's accountability and responsibility (influenced by fear and wanting to "do good"). (3) *Selfhood* - the conflict between the woman's self-assertive behavior in pursuing her needs AND the midwife's professional behavior - the woman as self-expert *versus* the midwife's professional identity	Values conflict<br>Individuals' values and context<br>Responsible to woman<br>Accountable for action<br>Woman responsible for choice<br>Justice<br>Do good<br>Identify own values<br>Midwifery identity<br>Professional attributes<br>Collaborative relationships	**Domain 1b, c, d, g**<br>**Domain 2e 2diii 4c**

Table 1. Data extraction table

11	**Foster et al., 2022[a]; Australia**	Midwives often experience moral compromise, leading to moral distress. Contributing factors include heavy workload, excessive paperwork, and service demands, which prevent them from practicing according to their core values and professional philosophy. The main issue identified is a poor hospital culture, dominated by hierarchical and biomedical values, which limits midwives' autonomy. The effects of moral distress vary among midwives and appear to be linked to their years of professional experience. moral distress - midwives cannot act in accordance with their moral or professional values. moral compromise - acting against ethical beliefs due to external constraints. Professional autonomy - not being able to make independent, evidence-based decisions consistent with midwifery philosophy.	Autonomy (midwifery)<br>EBP<br>Midwifery philosophy<br>Professional values<br>Individual values<br>system	**1 e 2e 4a<br>Domain 4**
12	**Foster et al., 2022[b], Australia**	Three core attributes of moral distress in midwifery are: concerns surrounding moral actions and inactions, meeting conflicting needs, and negative feelings and emotions.<br>Action referred to any instances where the midwife felt she was contributing to harm during her care of the woman. Inaction related more specifically to situations where the midwife did not speak up or challenge or aspects of care that they felt was detrimental to the woman or her interests. Conflicting needs: loyalties, priorities and values.<br>Conflicting priorities compromised the midwives' professional values of providing wholistic, safe and appropriate care in favour of meeting the organisational demands attending to a large volume of women Midwives obligation to the woman and their professional responsibility, specific circumstances, such as disagreeing with the rationale for the termination, were a cause of distress	Organizational demands<br>Individual values<br>System conflict<br>professional responsibility<br>Obligation<br>Holistic care<br>Loyalties and priorities<br>Advocacy	**Domain1<br>2diii<br>4c**
13	**Getu et al., 2025, Ethiopia**	The findings highlight the moral complexity midwives encounter in balancing professional responsibilities, patient rights, and contextual constraints. They manage ethical dilemmas through consultation and negotiation strategies and look for guidance from colleagues. Ethical dilemma - reflect complex interactions between personal beliefs,	Responsibility<br>System structure<br>Collegiate support<br>Conflict resolution<br>Personal values	**Preamble knowledge<br>1c, 2a, 2d 2e**

Table 1. Data extraction table

		professional ethics, institutional structure, and patient rights.<br>Autonomy and accountability - essential for promoting ethically sound and respectful maternal care, especially in settings with limited resources and cultural diversity.	Midwifery philosophy<br>Patients rights<br>Accountability<br>Autonomy (mid)<br>Respectful care<br>Cultural sensitivity<br>Resources	
14	**Hallgren et al., 2005, Sweden**	The findings highlighted both ethical and unethical ways in which midwives related to women and their partners during childbirth. Results of the analysis showed that ethical practice was reflected in relational behaviours characterized by presence, responsiveness, and respect, while unethical practices involved distance, lack of attentiveness, or failure to acknowledge parents' vulnerability. These findings were interpreted in light of Løgstrup's relational ethics, emphasizing the ethical responsibility inherent in midwives' relationships with women and their partners during childbirth.	Responsiveness<br>Respect<br>Presence<br>Relational ethics<br>Responsibility to women	**Domain 2, 3a b, f, 1b**
15	**Haseli et al., 2024, Iran**	The main findings revealed that midwifery students' experiences regarding threats to women's dignity during childbirth included professional incompetence, abuse of power imbalance, an exclusive focus on physical health with neglect of mental well-being, and structural issues within the system.<br>Professional incompetence reflected non-evidence-based interventions, the normalization of disrespect and abuse by healthcare providers, and the justification of violence during labor and delivery<br>Dignity - Insufficient professional competence among midwives, such as a lack of awareness of professional ethics and reliance on outdated care practices, leads to non–evidence-based interventions, the normalization of disrespect, and the justification of violence during childbirth as routine care.<br>The abuse of power imbalance was manifested through five critical sub-themes; The commanding tone during the care. Humiliation of the mother. Beating the mother. Discrimination.	Holistic care<br>Power balance<br>Structural system issue<br>Dignity<br>Competence<br>EBP<br>Respect<br>Dignity<br>Obstetric violence	**Domain 1e**<br>**Domain 1d**<br>**Domain 3f**<br>**Domain 2d**<br>**3a**

Table 1. Data extraction table

		Structural problems of the system reflected high workload and fatigue among staff, inappropriate environments, a lack of dignity in policies, processes, and laws, and the facilitation of disrespect and abuse in teaching hospitals.		
**16**	**Hazal et al. 2022**	Ethical dilemmas faced by these midwives while working in delivery rooms were mainly caused by hierarchy - Limitations by medics to the scope of practice of a midwives seen as a threat to midwifery ethics differences of approach to the birth process (105) – mainly unnecessary medicalisation hospital management (86) – mainly hierarchical (doctor or management) pressures, unable to preserve dignity and lack of resources Communication (21) – mainly conflict with colleagues differences in ethical approach (28) – mainly experience made for easier decision making & difficult decisions were upsetting – with feelings of helplessness	System / power imbalance / medicalisation<br>scope of practice<br>midwifery philosophy<br>dignity<br>Conflict resolution with colleagues<br>Preservation of women's dignity<br>Resources<br>Right to decision making	**1a, 1d, 1e, 1f**
**17**	**Honkavuo, 2022**	main theme is a human and caring factor in the midwifery students' Bildung* process on ethical competence.<br>Four subthemes: supporting through trust and responsibility – teachers, supervisors, peers. supporting through dignity and respect – of diversity in women/ gender-diverse people supporting through truthfulness and justice – of the taught theoretical content and supporting through dialogue and reflection<br>*Bildung describes an inner identity for formation of becoming a professional midwife. caring culture that permeates ethos	Supporting trust<br>Responsibility<br>dignity and respect for women<br>Truthfulness<br>Justice<br>Workplace culture<br>Education<br>Midwifery Reflection<br>Diversity<br>Caring culture	**1a, 1e, 2f, 3a, 3f, 4b**
**18**	**Khajehpour, 2024**	However, the ethical climate dimension of supervisors was the most positive among the studied participants, with all midwives reporting that they received respect and support from supervisors.<br>the treatment process of instructors and staff, with instructors showing a more client-centered perspective in the treatment process due to the nature of their academic role in the clinical environment.<br>atmosphere of cooperation and mutual relations between midwives and gynecologists in Iran is at a low level, doctor-centeredness and their superior prestige. Midwives not able to practice in delivery ward	Peer collegiality and respect<br>Woman-centred care<br>Interprofessional collegiality<br>Scope of practice<br>Power imbalance<br>Hierarchy /system<br>Midwifery autonomy	**1d, 1e, 1f, 3f**

		as they' – Limitations by medics to the scope of practice of a midwives seen as a threat to midwifery ethics		
19	**Khatoon s et al, 2025**	Deliberating ethical issues is an interactive way of learning. Dilemma Method of MCD improved midwives' knowledge and practice regarding respectful maternity care.<br>Also mention in discussion about impact of power/ hierarchy/ medics on ethics. 'A difference of opinion between a physician and a midwife can create a dilemma. The power hierarchy within medical teams can create challenges for midwives when resolving conflicts with doctors who hold higher positions. This can lead to stress and inappropriate interactions with patients.	Respectful maternity care<br>Midwifery knowledge<br>Power balance<br>System / medicalising / hierarchy<br>Midwifery philosophy<br>Interprofessional collegiality<br>Conflict resolution	**1c, 1d, 1f, 1g, 2c, 2d, 2e, 3e, 4c**
**20**	**Kiani, 2025**	ACEM-42 is a valid and reliable tool to assess adherence to codes of ethics in midwifery practice. The tool included these topics: Professional commitment – competent, awareness of ethical responsibility, respect dignity, Service – Evidenced based practice, adheres to law, safety, responds to crisis, Women-centred care – privacy, confidentiality, empathy, respect, provide information, quality care, woman's wishes, time, non-discrimination. Colleagues – cooperation and respect, reports non -compliance, Self – training self-monitoring /advancing the profession	Competence<br>Dignity<br>Respect<br>EBP<br>Crisis<br>Law<br>Safety<br>Woman-centred care<br>Privacy<br>Confidentiality<br>empathy,<br>provide information, quality care,<br>time,<br>non-discrimination<br>collegiality<br>reports non -compliance,<br>continuous education 'advancing the profession	2a 2d<br>Preamble 1, 3<br>1a 1e<br>2e<br>2d<br>2d iii
**21**	**MacLellan, 2014**	Women are vulnerable during childbirth and value relational aspects of care. The Ethic of Care approach equalise the relationship between midwife and woman, enabling relationship building and helping	Relationship<br>Respect<br>System	**1a**<br>**2diii**<br>**4a**

		midwives fulfil their professional responsibilities. Ethic of Care as a feminist ethical framework that centres relationships, and mutual respect. Relational balance between institutional demand and women's needs. Midwifery identity and practice being compromised without institutional acknowledgment of a distinct ethical approach.	Responsibility<br>Midwifery identity (philosophy)<br>Care ethics	
**22**	**Marsh et al., 2020**	- Midwives who care for women whose babies are removed at birth report it as one of the most distressing areas of contemporary clinical practice. Furthermore, they report feelings of guilt, helplessness and betrayal of the midwife–mother relationship. Many of the midwives in this study state that these experiences stay with them for a long time, far more than more joyful aspects of their role. Moral distress as a central ethical concept experienced when midwives' professional and emotional values conflict with institutional or legal requirements.	Midwifery philosophy<br>Personal values<br>Institution /system<br>Responsibility<br>Relationship	**4a 2c 2dii**<br>**1a**
**23**	**Martin- Badia, 2021**	Obstetric violence infringes on the main bioethical principles - Non-maleficence, Beneficence, Autonomy, and Justice.<br>- Humanization of care: Ethical midwifery practice requires recognizing women as biopsychosocial beings and promoting respectful, relationship-centered care. Beyond whether it is called violence or not, what matters from an ethical perspective is that, as long as women have such negative experiences during pregnancy and childbirth, obstetric care needs better humanizing.	Obstetric violence<br>Bioethical principles<br>Humanzing birth<br>Dignity<br>Physiological birth<br>Respect<br>Holistic care<br>Relationship	**1a, 1b, 1d2d**
**24**	**Meddings and Haith-Cooper, 2008**	Difficulties applying western bioethical principles - Autonomy, Beneficence and Non-maleficence, and Justice in cultural care<br>- Training in interpreter use, cross-cultural communication, and cultural humility is essential to support ethically appropriate, woman-centered care. To gain informed consent, midwives should assess whether a woman has a full understanding of the choices available. Surely this is impossible when the woman speaks little or no English.<br>- Culturally insensitive services do not respect the principle of justice.<br>- Rather than remaining 'culturally neutral,' it is essential to be culturally sensitive to meet their needs. Education and training are	Conflict with Autonomy, Beneficence and Non-maleficence, and Justice<br>Informed consent<br>Cultural sensitivity /cultural safety<br>Ensure choice is understood<br>Information provision<br>woman-centred care.	**2d iii**<br>**3g**<br>**2b**

		required to prepare service providers and midwives for a culturally diverse maternity population.		
**25**	**Megregian, 2016**	There is considerable variation in ethics education in terms of content, format, and evaluation among accredited midwifery education programs in the United States. Only 7 (28%) programs offer ethics as a stand-alone class. The majority of programs include the ethical concepts of informed consent, shared decision making, and effective communication.<br>Ethical principles in education: Programs consistently include informed consent, shared decision making, communication, privacy/confidentiality, and professional codes of ethics.<br>Midwifery ethics perspective: The study highlights the potential for developing a distinct midwifery ethic grounded in the principles of woman-centred care, advocacy, and shared decision making.	Distinct midwifery ethics<br>informed consent,<br>communication,<br>privacy/confidentiality,<br>woman-centred care,<br>advocacy,<br>shared decision making,	**3b**<br>**1c**
**26**	**Megregian et al., 2020**	Midwives described a range of professional ethical dilemmas, including challenges related to negotiating strained interprofessional relationships and protecting or promoting autonomy for women. Ethical dilemmas were identified by the theme of unease, a sense of distress that was expressed in three subthemes: uncertainty of action, compromise in action, and reflecting on action. Learning about ethics and ethical dilemmas occurred, for the most part, outside of the classroom, with the majority of participants reporting that their midwifery program did not confer the skills to identify and resolve ethical challenges.<br>Conclusion: inclusion of midwifery-specific ethics content in their curricula and in interprofessional ethics education. Reflection and self-awareness of bias were identified as key components of understanding ethical frameworks.	Interprofessional relationships<br>Women's autonomy<br>Uncertainty of action- right and wrong<br>Compromise of action -= unable to act<br>Moral distress<br>Autonomy<br>Informed consent<br>Midwifery ethics<br>Reflection	**1c**<br>**1e**<br>**3b**
**27**	**Megregian et al., 2021[a]**	Of the 12 statements on key content of ethics education, midwives emphasized that content promoting an understanding of shared decision-making is essential for inclusion. Of the statements regarding competencies, learning outcomes, and teaching strategies, 20 of 21 statements met consensus, including those related to shared decision-making and ethical decision-making, as well as attributes such as	shared decision-making<br>reproductive justice<br>compassionate care,<br>respectful care<br>courage<br>empathy	**1b**<br>**3f**<br>**4a**<br>**4b**

Table 1. Data extraction table

		compassion and courage. students promote effective decision-making, through an understanding of the elements of informed consent and refusal and skill in the shared decision-making process", achieved the highest mean score. As for key outcomes, "Students demonstrate behaviors consistent with compassionate care, including courage, empathy, honesty, kindness, and astute listening"		
**28**	**Megregian et al., 2021[b]**	Primary themes and associated subthemes: 1) current experiences and identified needs, 2) the preceptor dilemma, with subthemes the critical role of modeling ethics and powerlessness within interprofessional conflicts, and 3) complicated relationships: advocacy, autonomy and choice. Students reported their reliance upon the clinical preceptor and their clinical experiences with women, rather than any classroom activity, for providing the day-to-day learning of ethical behavior, interprofessional communication, and how to recognize, negotiate, and cope with ethical dilemmas.<br>The students identified interprofessional conflicts as a topic rife with ethical challenges and lacking in strategies for negotiating or resolving. The students in all focus groups consistently identified situations around informed consent and refusal as common ethical dilemmas they faced	ethics learning<br><br>shared decision-making<br><br>informed consent and refusal<br><br>advocacy<br><br>autonomy<br><br>interprofessional communication	**1c**<br>**1e**<br>**1f**
**29**	**Mizuno, 2011**	The results of this study revealed that midwives were torn between the roles of midwife and Termination of pregnancy (TOP) caregiver. Two major themes: 1) Experiences of midwives involved in both assisting in childbirth and care after TOP described by three theme clusters: (i) confusion about care of babies and aborted fetuses (ii) inability to provide adequate care for women in both situations who had different needs; and (iii) establing emotional control. 2) Professional awareness and attitude as a midwife. This theme described midwives' awareness and professional attitude in three theme clusters: (i) consistency with the midwives' professional principles; "It's the woman's decision. I'm there to help her. So that's what I do . . . and I do it for the woman; I'm providing a service for her.	Conflicting philosophies individuals' values and midwifery philosophy<br><br>Care conflict<br><br>professional confusion moral distress<br><br>conscientious objection<br>professional identity	**1c**<br>**1g**<br>**2c**<br>**2f**

Table 1. Data extraction table

		That's what I'm there for (ii) suppression of feelings in relation to the aborted fetus; and (iii) previous and current professional identities"		
**30**	**Newnham and Kirkham, 2019**	A care ethics approach would enable us to turn our perspective around and value relationships and caring first and foremost, highlight the unethical nature of regimented institutional birth and the damage caused by midwives' busyness and their requirement to push women through the system argue that this concept of autonomy is often annulled in the maternity field. Midwives and doctors are expected to place allegiance to hospital policy or cultural practices over respect for the wishes and needs of women. Suggest that a focus on care ethics has the potential to remedy these problems, by making power relationships visible and by prioritizing the relationship above abstract ethical principles.	care ethics,<br><br>relational care<br><br>autonomy<br><br>informed consent<br><br>moral/ethical climate of organizations,	**1a**<br>**1c**<br>**2d**<br>**2c**
31	**Oelhafen and Cignacco, 2020**	The core findings of the study are that ethical issues related to asymmetries of power and authority (e.g., lack of respect or inadequate staffing) are the most frequent causes of negative consequences (such as fatigue or job attrition), which serve as indicators of moral distress. Conversely, a positive ethical climate (characterised by respect and interprofessional trust) was identified as the strongest predictor associated with a reduction in these negative consequences. Self-assessed assertiveness also significantly reduced negative consequences. The most frequent problems were the patient receiving conflicting information from different health professionals, caesarean sections, or other interventions without a medical reason and inadequate staffing.	Competence<br>Transparent Information provision<br>Physiological birth<br>Medicalization<br>Respect<br>interprofessional trust<br>Advocacy	**2b, 2e 1e**
32	**Oelhafen et al., 2017**	Students perceive ethical problems as contrary to what they learnt in training, client autonomy seems to be limited, e.g., when a woman is against the induction of labor, and many decisions seem to be hierarchically driven as opposed to evidence-based. In this constellation, some students feel helpless and wonder what it helps to know better if they cannot change anything about the current situation. Good communication skills are essential in behaving in an	Communication skills<br>Ethics education<br>Midwives provide education to other midwives and students<br>Women's autonomy<br>Reflection<br>Collegial support (debriefing)	 **1e, 3a, 3e 3f**

Table 1. Data extraction table

		ethical manner. Good student support from experienced midwives lead to increased ethical competence in clinical settings. Students need more moral courage and should be able to ask critical questions Briefing and debriefing challenging situations is helpful but noted to be difficult to do in some clinics. Midwives stated they needed further education in ethics in order to support midwifery students appropriately.		
33	**Oelhafen et al., 2019**	Moral competency: Midwives may decide not to participate in activities for which they hold deep moral opposition; however, the emphasis on individual conscience should not deprive women of essential health services. Midwives with conscientious objection to a given service request will refer the woman to another provider where such a service can be provided. Moral problems in general themes:<br>- Autonomy and trust in building relationships<br>- Differing interprofessional values<br>- Institutional limitations of patient autonomy<br>- Hierarchical structures limiting midwife's autonomy<br>The midwives discussed moral competences and created an extensive list that can be considered: Competences to promote patient autonomy- Empower women and families to self-determination. Midwives need empathy and withhold their own opinions.	Midwifery relationships<br>Autonomy/ self determination<br>Midwifery philosophy<br>Collegial conflict<br>Institution / system<br>Autonomy (midwife)<br>Medicalisation<br>Own values<br>Empathy<br>Competence<br>Conscientious objection<br>Conflict resolution<br>Advocacy	**1a**<br>**3b**<br>**4a**<br>**4d**<br>**2a**<br>**2d iii**<br>**2b**
34	**Pezaro et al., 2025**	The authors argued that evolving midwifery language is not just a social adjustment but a professional responsibility necessary to maintain public trust and meet established ethical standards of practice. This is shown by discussions on how 'sexed language' causes clinical hard, violating the bioethical principle of non-maleficence. The principal of justice requires equal healthcare access for all birthing people therefore inclusive communication is a professional duty to upload reproductive justice. Midwives should move beyond tradition bioethics towards care ethics or queer bioethics to properly respect the bodily autonomy and individualised needs of a diverse population.	Minimise harm<br>Midwifery philosophy<br>Inclusive language<br>Reproductive justice<br>System/patriarchy<br>Care ethics<br>Queer ethics	**2d i, ii, iii**<br>**4a 4c**
35	**Ramsayer and Fleming, 2020**	This article shows that the decision to provide or object to abortion services remains ethically very complex because arguments exist both for and against its provision. Being with women can be interpreted	conscientious objection<br>scope of the midwife<br>respectful maternity care	**2b**<br>**1f**<br>**4d**

		differently and individual situations of care are multifaceted. Conscientious objection to abortion services is a highly contentious issue that has an overall importance to midwives. Noting that decisions are individual,<br>may change or may be situationally dependant; a definitive position of midwives for or against conscientious<br>objection cannot be assumed. Midwifery ethics centre on the philosophy of 'being with women', the protection of moral integrity through conscientious objection and the tensions between professional duties and personal values. Developing moral competence	'with woman'<br>Professional duties<br>Personal values<br>Moral competence	**1a**
36	**Rost, Montagnoli, and Eichinger, 2025**	Moral distress among midwives arises from conflicts between external constraints and personal moral standards, reflecting limited moral agency. Eight clusters of causes were identified: 1) Societal disregard, 2) Contemporary birth culture, 3) Lack of resources, ,4) Institutional characteristics, 5) Interprofessional hierarchies, 6) Interpersonal mistreatment of service users, 7) Defensive practice, and 8) Challenging care situations These conditions "undermine professional autonomy, dehumanize care, and erode midwives' moral integrity." (p.1394)<br>• Autonomy, constrained by hierarchy, policy, and law.<br>• Justice, inequities in resources and social recognition.<br>• Dignity, compromised by dehumanised and disrespectful care.<br>• Moral integrity & agency internal conflict, helplessness.<br>• Ethical reflection, need for moral communities and<br>• Non-maleficence, defensive practice and over-intervention harming trust and care quality.	Autonomy (midwives)<br>Autonomy (midwives)<br>Midwifery philosophy<br>Resources<br>System / institution<br>Mistreatment<br>Defensive practice<br>Respectful dignified care<br>Ethical reflection	**4a4d4e**<br>**1a 2d, 2d iii**<br>**1d**<br>**1g**<br>**3e**
37	**Schorn, 2023**	Midwives are ethically obligated to respect women's autonomy even when there is conflict or disagreement about care. Midwives must balance ethical principles (autonomy, beneficence, nonmaleficence, justice, veracity) with legal obligations to avoid abandonment. Recommendations: create clear practice policies; ensure documentation; improve communication; provide guidance on legal/ethical considerations; support midwives' boundaries while ensuring client safety The clinician must honor the client's refusal of	autonomy vs beneficence/ nonmaleficence.<br>Professional boundaries & self-protection.<br>Respect, communication, and therapeutic partnership.<br>Justice in fair treatment of clients.<br>Veracity in counseling.	**1e**<br>**1g**<br>**2diii**

		treatment even though it may conflict with the clinician's education and ethical obligation to promote health and save life. A case sample is used. Although the clinician supports the ethical principle of patient autonomy, the desire to practice in a beneficent and nonmaleficent manner in regard to the mother and fetus may put these principles at odds. The clinician's ethical desire to promote wellness and support client autonomy conflicts with the client's right to be free of unwanted medical treatment. The clinician must honor the client's refusal of treatment even though it may conflict with the clinician's education and ethical obligation to promote health and save life	Compassion.<br>Women's autonomy<br>conflicting obligations	
38	**Simbar M., et al., 2023**	Protecting physical privacy (draping, separate rooms, sound control) is an ethical obligation to preserve dignity and confidentiality.<br>Midwives recognize that clients' decision-making rights are often undermined; ensuring choice/informed consent is an ethical priority.<br>Midwives emphasize non-maleficence: invasive procedures should only occur with clinical indication to prevent harm and uphold midwifery values.<br>Midwives report lack of recognition and unfair allocation of credit/pay, this undermines professional status and autonomy.<br>Continuous education in ethics is necessary to support ethical practice and reduce moral distress.<br>Adequate staffing, space, and equipment are ethical necessities to provide safe, dignified care and prevent moral compromise.	Privacy<br>Confidentiality<br>Empathy<br>Autonomy<br>Fairness<br>Reflexive<br>Collegiate<br>Continued Education<br>Safe environments<br>Resources<br>Relationship<br>empathy, autonomy, fairness, self-evaluation, teamwork, continuing education, and safe environments.	**2c**<br>**1c**<br>**1e**<br>**3f**<br>**4b**
39	**Thachuk, 2007**	The midwifery model of informed choice is a more ethical, relational, and autonomy-enhancing approach than the medical informed consent model. Midwifery's holistic, continuity-based, woman-centered care promotes self-trust and supports autonomy, particularly for women who experience social oppression.<br>Recommendations: preserve midwifery values within regulation; maintain continuity of care; integrate relational autonomy principles in bioethics and practice; recognize social structures influencing women's decisions. Informed choice vs informed consent	Informed Choice, and Reproductive Autonomy:<br>Relational autonomy vs atomistic autonomy.<br>Informed choice vs informed consent.<br>Power dynamics in medical vs midwifery models.	**1c**<br>**2b**<br>**3b**<br>**1d**<br>**4c**

		"The process of informed consent as it stands… does not respect patient autonomy to its fullest extent."	Continuity of care as ethical practice.<br>Midwife client relationship & shared decision-making.<br>Ethical commitment to empowerment and non-paternalistic care.	
40	**Thompson F., 2002**	Interpretation of autonomy and informed consent have distinct context in childbearing compared to biomedicine (see quote in left column p. 529). Childbearing people should enter the setting with full autonomy (perhaps unlike a sick / dying person) and the childbirth context means although she is likely to have an ability to follow the cues of her own body, she is more susceptible to having this overridden by institution.<br>Codes offer little guidance for transforming principle into practice. Morality of relationships not addressed.<br>Ethical midwifery practice therefore needs to recognise the full (ie not reduced by illness) self-determination of childbearing people and focus on how to sustain it. The codes assume midwives to be accountable, autonomous and advocate, but work settings often to not actually permit these. Most significant implication for midwifery practice is a required change of focus to 'prime' relationship not abstract principles.	Autonomy – context<br>Informed consent<br>Maintain autonomy<br>Midwifery autonomy<br>Accountability<br>Advocacy<br>Primacy of relationship	**Domain 1**<br><br>**Domain 2 e, f**<br><br>**Domain 4, b**
41	**Thompson F., 2003**	Only ethically adequate if the practitioner also considered context, individual rights, virtues of personal character & relationship.<br>A conflict of values between workplace/service provider and personal ethics accompanied descriptions (themes) of power 'over', paternalism, lack of support for the birthing woman, and restriction or prevention from exercising professional midwifery judgement.<br>The midwives most commonly dealt with the conflict by becoming 'silenced' or subservient to medical authority, and by protecting the birthing woman by placating the 'system worker'.an adequate ethical response is one that redresses the imbalance of power within relationships. ethic of engagement reflects feminist virtue ethics. the	Balance Power<br>Relationship<br>Feminist ethics<br>Conflict with system<br>Human engagement	**Domain 1 (all)**

		disembeddedness and disembodiment of bioethics produces a conflict of values for both mothers and midwives in the maternity practice setting. Human engagement between individuals is a more appropriate ethical response		
42	**Thompson A., 2004**	Key ethical concepts in childbirth are: Choice, control, autonomy. Fetus as 'silent' client – best interests may conflict with that of the mother. To teach ethics, midwives need to: Know themselves, know their students, know their subject, know their clients. Ethics best taught by 'doing' (rather than theory). Use of codes (eg ICM) can be useful.<br>Examples of such structural constraints include,<br>● Lack of access to reproductive health care<br>● Injustice in the way in which services and benefits are administered<br>● Discrimination against women and girls at all levels: economic, cultural, and educational<br>be attentive to the values and beliefs of others—clients or colleagues— in a word, to be respectful of the "otherness	Choice,<br>control,<br>autonomy.<br>Mothers' baby dyad rights<br>Midwives know themselves<br>ICM Codes<br>Attentive	**Domain 1**<br>**Domain 3**<br>**1c**
43	**Thomspon J.B., 2004**	Focuses on the interface of values, ethics, and human rights in providing midwifery care to young girls and women throughout their lives. This interface constitutes a human rights framework for midwifery care that respects all persons, promotes informed decision making, takes measures to prevent any acts of violence or discrimination against clients, maintains privacy, and promotes safety for all seeking midwifery care. A human rights framework for midwifery care—a model supported by codes of ethics, the midwifery philosophy of care, and standards of practice	Values<br>Human rights<br>Respect<br>Autonomy<br>Prevent violence<br>Maintains privacy and safety<br>Midwifery philosophy<br>Standards of practice	
44	**Tömmel T. N., 2024**	Feminist care ethics most important philosophical source of relational understanding of autonomy. Author CONFLATES obstetric and midwifery care as if the same informed consent process alone cannot do justice to ethical claims of women in the course of reproductive decisions - a relational understanding of autonomy is essential for the ethics of obstetrics and midwifery.	Autonomy<br>feminist ethics<br>Relationship primacy for consent and autonomy<br>Care ethics<br>Obstetric violence	**Domain 1: a, b, c, d**<br><br>**Domain 4: b, c, d**

		obstetric violence occurs when relationality in birth is violated.<br>Care ethics emphasis on attentiveness, receptivity and responsivity of the care giver cannot be underestimated in its importance for midwifery care ethics<br>Moreover, respecting women's autonomy in childbirth means accepting her medically inadvisable choices	Justice	
45	**Tuming N. E. and Zolkefli Y., 2024**	Two broad themes identified: (1) Adequacy in knowledge and (2) Between expectation and reality. The connection between students' ethical education & experience with ethical concerns in the clinical setting. Second theme shows the significance that students attributed to ethical care – which comprises several aspects, including attitude, verbal and non-verbal communication and professionalism. When probed about situations that demonstrate good ethical judgement, most students highlight the significance of providing informed consent as one example. Provide information, upholding privacy and dignity.	Attitude<br>Advocacy<br>Informed consent<br>Communication<br>Attitude<br>Knowledge for consent<br>Consent<br>Privacy ad dignity	**Domain 1, e**.<br>**3b**
46	**van der Waal R. and van Nistelrooij I., 2025**	Most mothers in this study understand themselves to have intuitive or bodily knowledge on how to birth, on how to be in a relation of care and responsibility with their child when pregnant, and as embedded in a community of care and responsibility. Mothers felt actively expropriated by the obstetric system of their capacity to birth: The most listed occurrences of violence in obstetrics in this study are: (1) obstetric racism; (2) epistemic injustice, mainly playing the dead baby card; (3) physical violence,<br>consisting of interventions without consent; (4) penetrative violence, that is, violence linked to, or reminiscent of, rape or sexual assault; and (5) unconsented and/or unwarranted, and/or unwanted vaginal examinations. In the quote from a midwife in training below, all five of these forms of violence are present in a situation wherein a vaginal examination is being done.	Mother responsible for outcomes<br>Women's autonomy<br>Power imbalance<br>Obstetric violence<br>Advocacy<br>System conflict<br>Abolitionist care ethics<br>Care ethics<br>Feminist ethics<br>Reproductive justice	**1c1d**<br>**2d iii**<br>**2d**<br>**4c**
47	**van der Waal R., 2022**	The emphasis on the maternal–fetal conflict in ethics, obstetric practice, but also in popular culture, as the ethical dilemma and	Women's autonomy<br>Relational ethics	**1c**<br>**1a**

		medical problematic of pregnancy, not only unjustifiably neglects other issues but also reproduces the severance of the relation between mother and child. For reproductive justice and emotionally and physically safe maternity care to become possible, both the reproductive and the midwifery relation must be radically re-imagined. With this contribution we hope to underscore the need for care ethics because of its traditional focus on relationality, and relational autonomy in maternity care, as well as to lay bare what inhibits the relationalities necessary for a truly care-ethical praxis. Furthermore, it aims to shine another light on debates concerning abortion and childbirth leading to ethical questions and problematics that differ from those more commonly raised, centering on interwovenness relationality, community and solidarity	Relationship<br>Care ethics<br>Society community<br>Emotional safety<br>Reproductive justice	**3c**<br>**1b**<br>**2d**
48	**Vincifori E., 2014**	Ethical concepts captured in the survey instrument: "Midwives acknowledge the centrality of the woman, the couple and the newborn …A. Empowerment (36.0%). B. Solidarity (0.0%) C. Attendance (22.0%). D. Health promotion (19.4%) . E. Holism (21.5%). The statement "Midwives' practice hinges on freedom and independence of the profession . . ." (EC) is related to: A. Competence (26.9%) B. Responsibility (16.1%) C. Autonomy (50.0%) D. Integrity (3.8%) E. Unconstraint (1.6%) Q9 Which one of the following terms, defined by the EC, would best describe Italian Midwives Professional Identity?<br>A. Responsibility (22.6%)<br>B. Care planning (43.5%)<br>C. Conscience Clause (3.8%)<br>D. Accountability (10.2%)<br>E. Information supply (16.7%)	Empowerment<br>Solidarity<br>Attendance<br>Health promotion<br>Holism<br>Competence<br>Responsibility<br>Autonomy<br>Integrity	**1a, b**<br>**3c**<br>**2a**<br>**3b**<br>**3a**<br>**3f**<br>**3e**<br>**1e**<br>**4d**
49	**Yanow S., 2021**	Codes are gounded in bioethical principles. Nonmaleficence, which refers to doing no harm, is an ethical principle requires that the midwife avoid harming the patient, which can be derived from action, lack of action, or lack of knowledge. Midwives have an ethical obligation to become knowledgeable about options counselling. Without gaining and sharing this knowledge, midwives may place their	Bioethics<br>Woman centred Information provision<br>Scope of the midwife<br>Justice – access to health care<br>Prevent Harm<br>Continuing education	**3b**<br>**4d**<br>**2b**<br>**3d**<br>**1c**<br>**2d**

		patients at risk of unnecessary harm and, in doing so, violate their ethical obligations to their patients. This ethical obligation; it requires action on the part of the midwife to benefit the patient. In the context of bioethics, justice refers to the need for health care to be fair in its provision.<br>Patient education and support can reduce patient risk, and as such represents a harm-reduction strategy.<br>Within midwifery ethics to promote access to health care and end disparities to make information about abortion pills and how they are used more widely available, and for midwives to participate in that information-sharing<br>In the context of bioethics, justice refers to the need for health care to be fair in its provision. Midwives are trusted sources of information and often have long-term relationships with members of the community. Patient education and support can reduce patient risk, and as such represents a harm-reduction strategy.	Conscientious objection<br>Ethical obligation<br>Advocacy	
50	**Zolala S., 2019**	Example of moral distress from survey included: Assist a physician who performs a test or treatment without informed consent; Assist a physician who performs pregnancy termination or caesarean without informed consent; Ignore situations in which pregnant women have not been given adequate information to ensure informed consent<br>Let medical students perform vaginal exam on parturient solely to increase their skill; Follow the physician's order not to tell the parturient the truth when she asks; Follow the physician's request not to tell the status of parturient with the family when they ask you<br>Work with physician who is not as competent as the parturient care requires; Follow the physician's order to inappropriate injection or more medication, in order to precipitate the vaginal delivery<br>Follow the physician's order to inject narcotics that is harmful or ceases the delivery; Follow the physician's order not to consult with the family for caesarean ; Work with physician who avoid doing caesarean because patient not able to pay money; Observe without taking action when the physician do not respect the parturient privacy	Informed consent<br>Information provision<br>Practice – interventions for the Hp not for the woman<br>Truth withholding / transparency<br>Competence<br>Normal physiology<br>Consent<br>Care – equity<br>Conflict with other Health professionals<br>Reporting Harmful practices<br>Safety<br>Privacy & confidentiality<br>Dignity<br>Scope<br>System/ institutional policy<br>Equity / fairness / resources<br>Non EBP	**1c 3b**<br>**2a**<br>**4c**<br>**3b**<br>**2d iii**

Table 1. Data extraction table

		Follow the physician's order not to terminate pregnancy for non-medical reasons; Follow the physician's order to delay hospitalization.		

Preferred Reporting Items for Systematic reviews and Meta-Analyses extension for Scoping Reviews (PRISMA-ScR) Checklist

SECTION	ITEM	PRISMA-ScR CHECKLIST ITEM	REPORTED ON PAGE #
**TITLE**			
Title	1	Identify the report as a scoping review.	1
**ABSTRACT**			
Structured summary	2	Provide a structured summary that includes (as applicable): background, objectives, eligibility criteria, sources of evidence, charting methods, results, and conclusions that relate to the review questions and objectives.	1
**INTRODUCTION**			
Rationale	3	Describe the rationale for the review in the context of what is already known. Explain why the review questions/objectives lend themselves to a scoping review approach.	2
Objectives	4	Provide an explicit statement of the questions and objectives being addressed with reference to their key elements (e.g., population or participants, concepts, and context) or other relevant key elements used to conceptualize the review questions and/or objectives.	3
**METHODS**			
Protocol and registration	5	Indicate whether a review protocol exists; state if and where it can be accessed (e.g., a Web address); and if available, provide registration information, including the registration number.	3/4
Eligibility criteria	6	Specify characteristics of the sources of evidence used as eligibility criteria (e.g., years considered, language, and publication status), and provide a rationale.	4/5
Information sources*	7	Describe all information sources in the search (e.g., databases with dates of coverage and contact with authors to identify additional sources), as well as the date the most recent search was executed.	5/6
Search	8	Present the full electronic search strategy for at least 1 database, including any limits used, such that it could be repeated.	6
Selection of sources of evidence†	9	State the process for selecting sources of evidence (i.e., screening and eligibility) included in the scoping review.	6
Data charting process‡	10	Describe the methods of charting data from the included sources of evidence (e.g., calibrated forms or forms that have been tested by the team before their use, and whether data charting was done independently or in duplicate) and any processes for obtaining and confirming data from investigators.	7
Data items	11	List and define all variables for which data were sought and any assumptions and simplifications made.	7
Critical appraisal of individual sources of evidence§	12	If done, provide a rationale for conducting a critical appraisal of included sources of evidence; describe the methods used and how this information was used in any data synthesis (if appropriate).	7

SECTION	ITEM	PRISMA-ScR CHECKLIST ITEM	REPORTED ON PAGE #
Synthesis of results	13	Describe the methods of handling and summarizing the data that were charted.	8
**RESULTS**			
Selection of sources of evidence	14	Give numbers of sources of evidence screened, assessed for eligibility, and included in the review, with reasons for exclusions at each stage, ideally using a flow diagram.	7
Characteristics of sources of evidence	15	For each source of evidence, present characteristics for which data were charted and provide the citations.	10
Critical appraisal within sources of evidence	16	If done, present data on critical appraisal of included sources of evidence (see item 12).	9
Results of individual sources of evidence	17	For each included source of evidence, present the relevant data that were charted that relate to the review questions and objectives.	9
Synthesis of results	18	Summarize and/or present the charting results as they relate to the review questions and objectives.	9,10
**DISCUSSION**			
Summary of evidence	19	Summarize the main results (including an overview of concepts, themes, and types of evidence available), link to the review questions and objectives, and consider the relevance to key groups.	9-14
Limitations	20	Discuss the limitations of the scoping review process.	15
Conclusions	21	Provide a general interpretation of the results with respect to the review questions and objectives, as well as potential implications and/or next steps.	15
**FUNDING**			
Funding	22	Describe sources of funding for the included sources of evidence, as well as sources of funding for the scoping review. Describe the role of the funders of the scoping review.	15

JBI = Joanna Briggs Institute; PRISMA-ScR = Preferred Reporting Items for Systematic reviews and Meta-Analyses extension for Scoping Reviews.
* Where *sources of evidence* (see second footnote) are compiled from, such as bibliographic databases, social media platforms, and Web sites.
† A more inclusive/heterogeneous term used to account for the different types of evidence or data sources (e.g., quantitative and/or qualitative research, expert opinion, and policy documents) that may be eligible in a scoping review as opposed to only studies. This is not to be confused with *information sources* (see first footnote).
‡ The frameworks by Arksey and O'Malley (6) and Levac and colleagues (7) and the JBI guidance (4, 5) refer to the process of data extraction in a scoping review as data charting.
§ The process of systematically examining research evidence to assess its validity, results, and relevance before using it to inform a decision. This term is used for items 12 and 16 instead of "risk of bias" (which is more applicable to systematic reviews of interventions) to include and acknowledge the various sources of evidence that may be used in a scoping review (e.g., quantitative and/or qualitative research, expert opinion, and policy document).

From: Tricco AC, Lillie E, Zarin W, O'Brien KK, Colquhoun H, Levac D, et al. PRISMA Extension for Scoping Reviews (PRISMAScR): Checklist and Explanation. Ann Intern Med. 2018;169:467–473. doi: 10.7326/M18-0850.
